# Cosmic silence and viral noise: transcriptomic crosstalk in *Caenorhabditis elegans* under simulated space conditions

**DOI:** 10.3389/fmicb.2026.1781245

**Published:** 2026-03-18

**Authors:** Ana Villena-Giménez, Esmeralda G. Legarda, Rubén González, Victoria G. Castiglioni, Santiago F. Elena

**Affiliations:** 1 Instituto de Biología Integrativa de Sistemas (CSIC-UV), Paterna, Valencia, Spain; 2 Viruses and RNA Interference Unit, Institut Pasteur, Université Paris Cité, Paris, France; 3 Department of Molecular Genetics, University of Toronto, Toronto, ON, Canada; 4 Santa Fe Institute, Santa Fe, NM, United States

**Keywords:** defective viral genomes, gene expression, host-pathogen interaction, microgravity, Orsay virus, radiologically shielded environments, stress response

## Abstract

**Background:**

Spaceflight environments pose unique physiological challenges due to al-tered gravity and radiation exposure.

**Methods:**

To investigate how these abiotic stressors interact with viral infections, we ana-lyzed the transcriptomic response of *Caenorhabditis elegans* acclimated to low-shear mod-eled microgravity (LSMMG) and radiologically shielded environments (RSE), after being infected with Orsay virus (OrV). Using RNA-sequencing, we characterized gene expres-sion profiles across both single and combined stress conditions.

**Results:**

Both LSMMG and RSE elicited distinct stress responses, including the modula-tion of oxidative stress, lipid metabolism, and immune pathways. The OrV infection alone induced robust transcriptional changes, but its impact was significantly attenuated when combined with abiotic stress, suggesting an antagonistic interaction. Notably, proviral genes such as *drl-1*, *fat-7*, and *hipr-1* were downregulated under RSE and LSMMG condi-tions, potentially impairing the viral replication. Gene ontology analyses revealed enrich-ment in immune effectors, RNA metabolism, and proteostasis-related pathways, particular-ly under RSE. Viral load and RNA2/RNA1 ratios were reduced in both stress conditions, indicating a shift in viral replication dynamics. Moreover, genomic diversity and defective viral genome formation were affected differentially, with stress conditions leading to in-creased genetic diversity and structural variation.

**Discussion:**

These findings suggest that acclimation to simulated off-Earth conditions primes the host for a dampened response to acute viral infections, potentially through re-source reallocation and transcriptional attenuation. This study provides transcriptomic insight into viral infections under space-relevant conditions, highlighting complex interac-tions between stressors and their implications for host-pathogen dynamics in extraterres-trial environments.

## 1 Introduction

Space exploration is accelerating, with future missions to the Moon and Mars poised to expose organisms to combined stressors, including altered gravity and non-terrestrial radiation fields. The absence of gravity or microgravity in low Earth orbits disrupts human physiology across multiple systems, notably musculoskeletal and cardiovascular function (Tanaka et al., 2017). The immune system is also affected: Astronauts exhibit altered lymphocyte distributions (Grove et al., 1995), reduced NK cell activity (Konstantinova et al., 1995), elevated proinflammatory mediators such as IL-6 and cortisol (Stein and Schluter, 1994), and depressed virus-specific T-cell responses (Crucian et al., 2013). Ground-based low-shear modeled microgravity (LSMMG) simulations recapitulate increased inflammatory tone and reduced T- and NK-cell function (Wu et al., 2024). Clinically relevant outcomes include viral reactivation during missions: Epstein–Barr virus (EBV), varicella zoster virus, and cytomegalovirus can increase in frequency, duration, and copy number even during short flights (Mehta et al., 2017). In a study involving mice flown to space, transcriptomic analysis reveals upregulation of virus-related pathways (e.g., prion disease, coronavirus disease, and hepatitis A, B, and C) (Zhang et al., 2024). However, findings under simulated LSMMG conditions are mixed: Kaposi’s sarcoma-associated herpesvirus (Honda et al., 2020) and EBV (Long et al., 1999) can remain latent, whereas latent retroviral transcription can be induced in human immune cells after ~ 25 h of exposure to LSMMG (Wu et al., 2024). Microbiome-associated viromes also appear dynamic: Phages and other potential viral pathogens within the human microbiota show increased activity during in-flight phases (Tierney et al., 2024). Two very recent studies have shown that LSMMG alters phage–host interactions by delaying infection and driving distinct evolutionary trajectories in both viruses and bacteria. In a study conducted on the International Space Station (ISS) (Huss et al., 2026), DNA phage T7 and *Escherichia coli* accumulated unique mutations that reshaped their coevolution and even produced phage variants with enhanced infectivity against resistant terrestrial strains. In a simulated LSMMG study (Rodríguez-Moreno et al., 2025), RNA phage Qβ showed reduced initial titers and repeatedly fixed a specific adaptive mutation, which improved infection dynamics only under LSMMG conditions. Therefore, the net impact of LSMMG on viral dynamics warrants further exploration.

Radiation exposure is a parallel challenge. On the ISS, annual effective doses (~ 110–180 mSv year^−1^) exceed those on Earth (~ 0.5–70 mSv year^−1^) (Restier-Verlet et al., 2021). Crucially, radiation composition differs: Space exposures include galactic cosmic rays, solar particle events, and trapped belt electrons/protons (Freese et al., 2016), while terrestrial exposure is dominated by telluric sources (e.g., radon), radioactive materials, and attenuated cosmic rays (Restier-Verlet et al., 2021). On Earth’s surface, ~85% of detected cosmic radiation comprises muons, which are low-linear energy transfer (LET), highly penetrating particles with high flux (Atri and Melott, 2011; Atri and Melott, 2014). These muons are largely absent in deep underground environments; intriguingly, studies in radiologically shielded environments (RSEs) within such facilities report increased radiosensitivity (Carbone et al., 2009), diminished cellular defense mechanisms (Fratini et al., 2015), and broad transcriptional reprogramming, including downregulated primary metabolism and upregulated immune and stress-response pathways (Zarubin et al., 2021). These observations have led to the hypothesis that the chronic muon flux contributes to normal physiological set points and that RSEs can perturb cellular homeostasis.

The nematode *Caenorhabditis elegans* is a well-established model system for space biology due to its ease of culture, rapid life cycle, compact size, and deep molecular annotation, including substantial human gene homology (Lai et al., 2000; Zhang et al., 2025). The discovery of the Orsay virus (OrV) enables host–virus studies in a whole-animal, genetically tractable system (Félix et al., 2011). OrV is related to the *Nodaviridae* family and has a small, bipartite, non-enveloped, positive-sense single-stranded RNA genome. Its RNA1 encodes an RNA-dependent RNA polymerase (RdRP), while RNA2 encodes a capsid protein (CP) and the *δ* protein, which facilitate non-lytic viral release (Yuan et al., 2018). A CP-δ fusion protein, generated by a ribosomal frameshift, plays a role in receptor binding and virus entry (Jiang et al., 2014; Fan et al., 2017). OrV is an intestinal pathogen transmitted orofecally, whose infections are generally mild, with limited effects on lifespan or reproduction in some *C. elegans* strains (Félix et al., 2011; Ashe et al., 2013).

In *C. elegans*, biotic and abiotic stressors can interact non-linearly. For example, ZnO nanoparticles or *Klebsiella pneumoniae* alone suppress reproduction, yet together show no effect (Cochran et al., 2023). Mild stress preconditioning can increase survival under subsequent severe stress, including diet-dependent cadmium resilience (Dölling et al., 2019). Crosstalk between abiotic stress and infection is bidirectional: OrV infection increases heat-shock resistance (Castiglioni and Elena, 2024), while heat stress enhances resistance to OrV (Huang et al., 2021), with overlapping transcriptional responses enriched for *pals*-family genes and intracellular pathogen response (IPR) modules (Huang et al., 2021; Zhou et al., 2019). OrV infection during larval development progresses through four distinct phases: Prereplication (0–6 hpi), exponential replication peaking at 12 hpi, moderate replication (14–36 hpi), and persistent residual infection (38–44 hpi) (Castiglioni et al., 2024). After the acute phase, a latent infection is established (Castiglioni et al., 2025).

We previously described complex interactions between OrV infection, simulated LSMMG, and RSEs (Villena-Giménez et al., 2025). In this previous study, we used a fully factorial experimental design examining how LSMMG, RSEs, and OrV infection, alone and in all combinations, influence physiological traits and viral load. Although RSEs radically affected viral accumulation dynamics, LSMMG had a minor effect. Both factors significantly impacted reproduction and morphology, with some effects magnified by viral infection. These results revealed how even partial modifications to Earth-like gravity and radiation levels can alter pathogen–host interactions. Other studies have shown that simulated LSMMG recapitulates spaceflight-like gene expression shifts in *C. elegans*, particularly in cytoskeletal, muscle, and metabolic pathways (Higashibata et al., 2016), including a core of ~118 LSMMG-responsive genes enriched for locomotion, morphogenesis, and cuticle biology, 44 of which correspond to 64 human orthologs (Çelen et al., 2023). In RSEs, *C. elegans* transcriptomes show upregulated sperm proteins and downregulated cuticle/collagen genes (Van Voorhies et al., 2020). However, the interplay between viral infection and the combined stresses of LSMMG and reduced muon flux remains unexplored.

In this study, we characterize the transcriptomic landscape of OrV infection in *C. elegans* acclimated to simulated off-Earth conditions, building on previous research (Villena-Giménez et al., 2025). To do so, we exposed larvae populations to simulated LSMMG using a random positioning machine (RPM), to RSEs in the Canfranc Underground Laboratory (LSC), and to OrV infection, each individually and in combination (LSMMG + OrV and RSE + OrV). As intergenerational stress responses can persist for multiple generations (Kishimoto et al., 2017), animals were acclimated for two generations to LSMMG or RSEs to capture long-term, rather than acute, abiotic-stress responses. Nematodes were inoculated immediately after hatching with a high-concentration OrV inoculum and collected 14 hpi, corresponding to the reported peak viral load (Castiglioni et al., 2024). Developmental stage was equivalent across all conditions at 14 hpi (Melero et al., 2025), with LSMMG and RSEs exerting significant but minor effects on development (Villena-Giménez et al., 2025). From the viral standpoint, this framework allows us to quantify how these abiotic stresses influence the accumulation of OrV and its diversity within hosts. Notably, the RSE conditions explored here align with environments characterized by “cosmic silence” and are relevant to future extraterrestrial niches such as subsurface Mars, icy moon oceans, or planets with dense atmospheres.

## 2 Materials and methods

### 2.1 *Caenorhabditis elegans* strains and culturing

*Caenorhabditis elegans* was cultured and maintained at 20 °C on nematode growth media (NGM) agar plates seeded with *Escherichia coli* OP50. ERT54 (*jyIs8[pals-5p: GFP + myo-2p:mCherry]X*), a transgenic strain with a genetic wild-type (Bristol N2) background that expresses GFP in response to intracellular infection (Bakowski et al., 2014), was used for all experiments. The more susceptible strain SFE2 (*drh-1(ok3495)IV;mjls228*) was used to produce OrV stocks.

To obtain synchronized animal populations, plates with embryos were carefully washed with M9 buffer to remove larvae and adults while leaving the embryos behind. The plates were washed again with M9 buffer after 1 h to collect larvae that hatched within that time span, and these were transferred to seeded NGM plates.

### 2.2 Low-sheared modeled microgravity simulation

Experiments were performed as previously described (Villena-Giménez et al., 2025). Briefly, an RPM (Yuri Gravity GmbH) was used to simulate microgravity conditions by selecting the zero-gravity mode. The *g*-force values were monitored using the RPM software and maintained at approximately 0.001–0.002 g throughout the experiments. The RPM was placed in an incubator at 20 °C. Control gravity conditions corresponded to standard surface gravity (1 g) in a side-by-side incubator at 20 °C.

Animals used in LSMMG experiments were acclimated for two generations, with studies conducted on the third generation and its progeny. Plates were sealed with parafilm to maintain their humidity.

### 2.3 Radiologically shielded environmental conditions

Experiments were performed in the LAB2400 at the Canfranc Underground Laboratory (LSC), located in Estación de Canfranc (Huesca, Spain), as previously described (Villena-Giménez et al., 2025). The measured integrated muon radiation flux in this facility is ~ 0.005 m^−2^ s^−1^ (Trzaska et al., 2019). For comparison, the muon radiation flux at sea level in the northern hemisphere is approximately 150 m^−2^ s^−1^ (Particle Data Group, 2022).

Thermoluminescent dosimeters sensitive to various sources of radiation are placed at different locations in the underground laboratory. In 2022, they showed an average dose rate of 0.71 ± 0.03 mSv year^−1^, compared to 1.36 ± 0.03 mSv year^−1^ in the above-ground laboratory (Hernández-Antolín et al., 2024). Regarding radiation produced by radon, a good ventilation system and a Radon Abatement System reduced radiation levels to 0.001 Bq m^−3^, substantially lower than the 200–280 Bq m^−3^ measured in the surface laboratory (Pérez-Pérez et al., 2022).

Nematodes used in RSE experiments were acclimated to this condition for two generations prior to experiments.

### 2.4 Viral stock preparation, virus quantification, and inoculation procedure

For OrV (strain JUv1580_vlc) stock preparation, SFE2 animals were inoculated as previously described (Castiglioni et al., 2024). In short, animals were allowed to grow for 5 d and then resuspended in M9 (0.22 M KH_2_PO_4_, 0.42 M Na_2_HPO_4_, 0.85 M NaCl, 1 mM MgSO_4_), allowed to stand for 15 min at room temperature, vortexed, and centrifuged for 2 min at 400 g. The supernatant was centrifuged twice at 21,000 g for 5 min and then passed through a 0.2 μm filter. RNA from the resulting viral stock was extracted using the Viral RNA Isolation Kit (NZYTech). The concentration of the viral RNA was then determined by RT-qPCR using a standard curve and normalized across different stocks (details below). Primers used for RT-qPCR are found in Supplementary Table S1.

For the standard curve, cDNA of JUv1580_vlc was obtained using AccuScript High-Fidelity Reverse Transcriptase (Agilent) and reverse primers at the 3′ end of the genome. Approximately 1,000 bp of the 3′ end of RNA2 were amplified using forward primers containing a 20 bp sequence encoding the T7 promoter and DreamTaq DNA Polymerase (Thermo Fisher Scientific). The PCR products were gel-purified using MSB Spin PCRapace (Invitek Molecular), and an *in vitro* transcription was performed using T7 Polymerase (Merck). The remaining DNA was then degraded using DNase I (Life Technologies). RNA concentration was determined using a NanoDrop spectrophotometer (Thermo Fisher Scientific), and the number of molecules per μL was determined using the online tool EndMemo RNA Copy Number Calculator^1^. Primers used for the standard curve are found in Supplementary Table S1.

For inoculation experiments, synchronized populations were inoculated by pipetting 60 μL of the viral stock on top of the bacterial lawn containing the animals. The normalized inoculum contained 2.6 × 10^7^ copies of OrV RNA2/μL. The efficiency of this viral stock (measured as the percentage of animals showing activation of the *pals-5p: GFP* reporter at 48 hpi) was 72 ± 3% (mean ±1 SEM, *n* = 5 plates with 44–48 animals per plate).

### 2.5 RNA extractions

*Caenorhabditis elegans* sample preparation and RNA extractions were performed as previously described (Castiglioni et al., 2024). Synchronized populations of 300 inoculated and control animals were collected at 14 hpi using PBS-0.05% Tween. Samples of inoculated animals were prepared in triplicate. The samples were centrifuged for 2 min at 1350 rpm, and the supernatant was discarded. Furthermore, two additional wash steps were performed before freezing the samples in liquid nitrogen. A total of 500 μL of TRIzol (Invitrogen) was added to the nematode pellets and disrupted using five cycles of freeze-thawing followed by five cycles of vortexing for 30 s with 30 s rest between cycles. Next, 100 μL of chloroform was added, and the tubes were shaken for 15 s and allowed to rest for 2 min. The samples were centrifuged for 15 min at 11,000 g at 4 °C, and the top layer containing RNA was then mixed with an equal volume of 100% ethanol. The sample was then loaded onto RNA Clean & Concentrator columns (Zymo Research), and the remaining steps of the protocol were carried out according to the manufacturer’s instructions.

### 2.6 Total RNA extraction and preparation for RNA-seq

Sample preparation was performed as previously described (Castiglioni et al., 2024). Library preparation and Illumina sequencing were performed by Novogene Europe^2^ using a NovaSeq 6,000 platform. A lnc-stranded mRNA-seq library method was used, including ribosomal RNA depletion, directional library preparation, 150 bp paired-end sequencing, and 6 Gb of raw data per sample. Novogene assessed the quality of the libraries using a Qubit 4 Fluorometer (Thermo Fisher Scientific), performed qPCR for quantification, and used a Bioanalyzer to evaluate size distribution.

### 2.7 RNA-seq and host data processing

The quality of the resulting FASTQ files was assessed using FastQC (Andrews, 2010) and MultiQC (Ewels et al., 2016). A preprocessing step using bbduk.sh was performed to remove adapters and trim read ends with low quality (trimq = 10). Then, reads were mapped to the reference genome of *C. elegans* using STAR (Dobin et al., 2013), with the genome of N2 Bristol and its annotations (GCF_000002985.6_WBcel235) downloaded from the NCBI as input.

To obtain the matrix of counts per gene and sample, the function summarizeOverlaps from the package GenomicAlignments (Lawrence et al., 2013) was used (strand-aware, for paired-end reads, with mode = “Union”). This matrix count was annotated using the dataset wbps_gene from parasite_mart, AnnotationDbi, and org. Ce.eg.db. Exploratory analyses, such as principal components analysis (PCA) and clustering in R, were performed and visualized after applying a variance-stabilizing transformation (VST).

We used DESeq2 (Love et al., 2014) for differential expression analysis, applying a filter of a minimum of 10 counts in at least three samples (the size of our groups). Factors of unwanted variation (*W*_1_ + *W*_2_) were previously estimated using replicate samples with the R package RUVSeq (Risso et al., 2014). The complete design was set as ~ *W*_1_ + *W*_2_ + virus + treatment + virus × treatment and was assessed using the Wald test. A likelihood-ratio test (LRT) was used to confirm the necessity of including the interaction term. The IHW package was used for the multiple testing procedure, and DESeq2 was used to shrink log_2_ fold changes (*FCs*). DEGs were considered significant at a Benjamini–Hochberg-adjusted *p*-value of < 0.05. In heatmaps, asterisks indicate the significance level.

### 2.8 Data visualization

Venn diagrams were generated using the R library VennDiagram, including only genes with an adjusted *p*-value of < 0.05 for each group. The same number of genes was used as input for subsequent functional analyses. Over-representation analysis (ORA) was performed using the hyperGTest method from GoStats (Falcon and Gentleman, 2007), with a cutoff of *p* = 0.05 and all genes from org. Ce.eg.db used as the universe.

Gene networks were constructed using Cytoscape v3.10.4 (Shannon et al., 2003), selecting the option “Full STRING network” with a confidence cutoff of 0.4 and no additional interactors. For community clustering, GLay from clusterMaker2 (Morris et al., 2011) was used.

### 2.9 Virus characterization

After preprocessing the FASTQ files, bwa-mem (Li, 2013) was used to map OrV reads. Normalized viral load was calculated by dividing viral read counts by host read counts. For sequence diversity, we used normalized Shannon entropy (*H_n_*) per position, calculated as described previously (Gregori et al., 2014). We also determined the number of positions in each sample that fall within the top 5% of all entropy values. For non-standard viral genome (nsVG) inspection, DVGfinder (Olmo-Uceda et al., 2022) was run using the metasearch mode for the two segments of the OrV genome separately, after sequences mapped to the host genome were removed. We applied stringent filtering criteria to the results, retaining only nsVGs with a minimum length of five nucleotides and supported by at least 10 independent reads in one or both of the algorithms implemented, ViReMa (Sotcheff et al., 2023) and DI-tector (Beauclair et al., 2018), thereby restricting the analysis to high-confidence events. We did not separate the data by the sense of the nsVGs. The abundance of nsVGs was normalized and expressed in terms of counts per 100,000 viral reads (viral counts per hundred thousand reads; VCPHT).

## 3 Results and discussion

### 3.1 Stress acclimation produces antagonistic transcriptional interactions with viral infection

**Figure 1:**
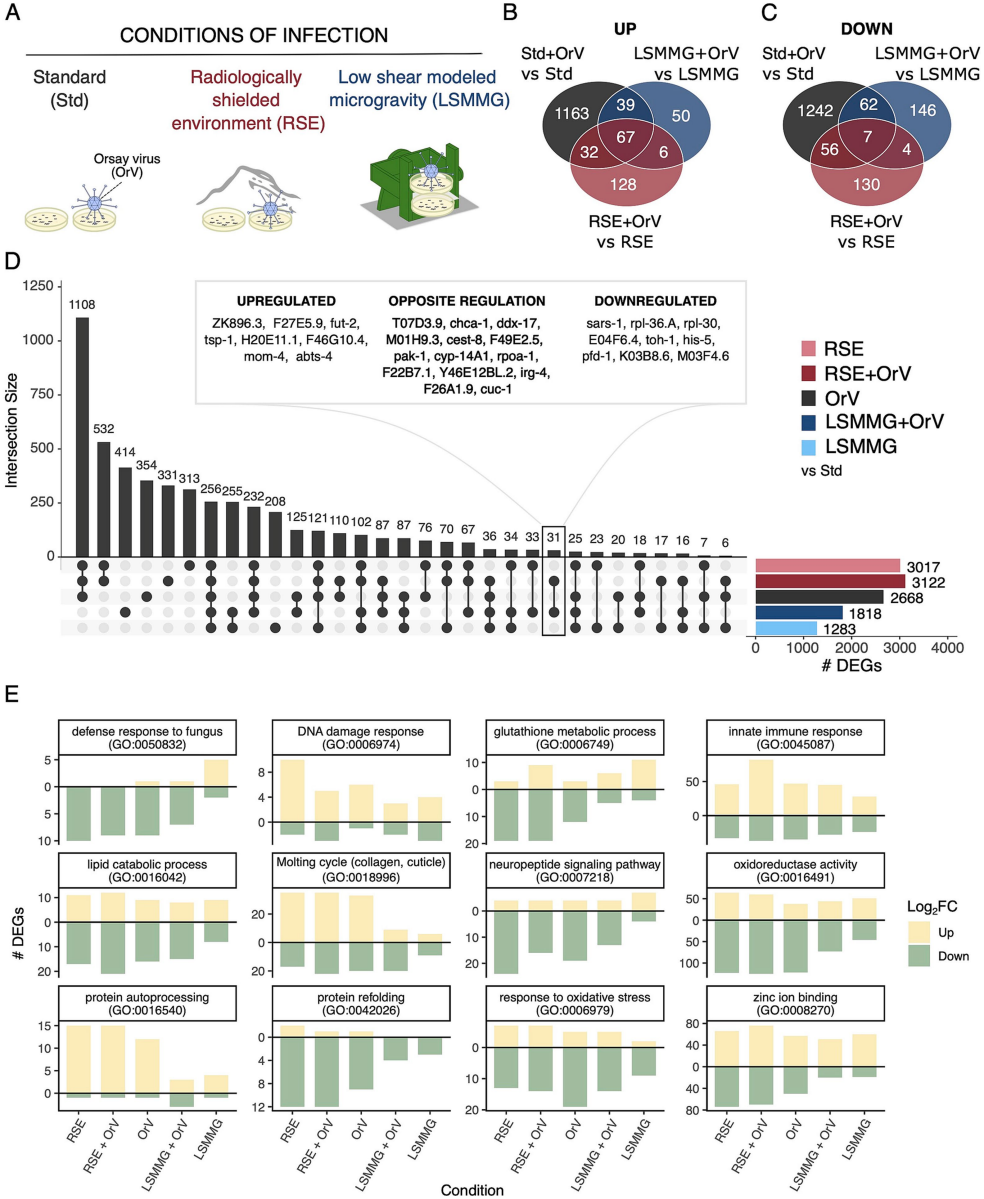
**(A)** Schematic representation of the experimental design. **(B,C)** Venn diagrams show upregulated and downregulated genes across infection conditions, based on contrasts between infected and non-infected samples within each condition. **(D)** Upset plot illustrates exclusive set intersections among physical and infection conditions compared to non-infected nematodes under standard conditions. The total size of each set is shown in the right bar plot, while all possible intersections are represented below, with their frequencies displayed in the top bar plot. **(E)** Comparison of infection conditions by GO categories, showing the number of differentially expressed genes associated with each term, using the same contrast strategy as in **(D)**.

We first quantified the effect of infection on the host transcriptional response within each background (Figure 1A and Supplementary Figure S1) using a general linear model (~ virus + treatment + treatment × virus). Across conditions, OrV elicited a robust core response (67 upregulated and seven downregulated genes; Figures 1B,C, Supplementary Figures S2A,B, and Supplementary Table S2), indicating a conserved infection program that persists despite environmental acclimation. GO enrichment analyses revealed that this program includes innate immune response, NAD-cap decapping, and cell wall-related processes (Supplementary Figure S2C). These cell wall terms likely reflect the host response to bacterial cell wall components, particularly disaccharide and peptidoglycan catabolic processes, suggesting a key role for bacterial peptidoglycans in the OrV response. These molecules, derived from bacterial food, act as digestive signals that drive metabolic adjustments to varied food environments (Hao et al., 2024). Previous studies have shown that the *C. elegans* food source modulates OrV infection (Vassallo et al., 2024; González and Félix, 2024).

To benchmark stress magnitude, we contrasted each treatment with non-infected nematodes under standard conditions (i.e., surface gravity of 1 g and radiation levels) (Figure 1D and Supplementary Figures S1G,H). RSEs and OrV alone produced similarly large transcriptomic responses (3,017 and 2,668 DEGs; adjusted *p* < 0.05), whereas LSMMG yielded fewer (1,283). When combined with infection, the overall response intensified under RSEs (3,122 DEGs) but was partially antagonistic under LSMMG (1,818). Interaction terms, indicating non-additive effects where combined stresses produce responses different from the sum of individual effects, were substantial: 1,940 DEGs for RSE × virus and 1,159 for LSMMG × virus, with 947 DEGs shared between the interactions (Supplementary Figure S2D). These patterns reveal a conserved coupling among stress pathways. GO summaries further highlighted recurring modulation of proteostasis (e.g., protein auto-processing and refolding), oxidative stress/oxidoreductase activity, lipid catabolism, and zinc binding, consistent with infection-driven proteotoxic load (Chen et al., 2017; Reddy et al., 2017), the lipid and zinc dependence of replication (Casorla-Perez et al., 2022), and spaceflight-associated lipid remodeling (Adenle et al., 2009) (Figure 1E). Notably, OrV alone elicited the most extensive oxidative stress response, which was attenuated by prior acclimation to RSEs or LSMMG, consistent with stress conditioning.

### 3.2 RSE-specific suppression of proviral genes and shared repression of lipid metabolism genes under RSEs and LSMMG

We next examined how RSEs and LSMMG affect the expression of proviral genes—host genes required for OrV infection (Supplementary Table S3). Compared to standard surface radiation, RSEs modestly upregulated *alg-1* (Supplementary Figure S2E; log₂*FC* = 0.573, adjusted *p* = 0.001) and downregulated *drl-1* (Supplementary Figure S2E; log_2_*FC* = −0.788, adjusted *p* = 0.007) and *hipr-1* (Supplementary Figure S2E; log_2_*FC* = −0.379, adjusted *p* = 0.005), three proviral factors that are essential during early OrV replication (Parker et al., 2007; Sandoval et al., 2019; Jiang et al., 2020). In parallel, compared to non-infected animals, OrV infection suppressed fatty acid regulators (*elo-1*, *elo-2*, *fat-6*, *fat-7*, *nhr-49*, and *nhr-80*) at 14 hpi (Supplementary Figure S2F), a pattern recapitulated under RSEs. In the comparison of RSE + OrV *vs.* RSEs, only *fat-7* exhibited a significant reduction (log_2_*FC* = −0.821, adjusted *p* = 0.002). Moreover, interaction terms were significant across this lipid module, indicating non-additive control and potentially highlighting the existence of a regulatory limit. Under LSMMG, *fat-6*, *fat-7,* and *nhr-80* were also downregulated (all adjusted *p* ≤ 0.009), with no significant changes observed in the comparison of LSMMG + OrV *vs.* LSMMG. Significant LSMMG × OrV interactions were detected for *elo-1*, *fat-7*, *nhr-49*, and *nhr-80* (Supplementary Figure S2F). Together, these results position lipid metabolism as a shared mechanism through which LSMMG and RSEs shape early infection dynamics and host fitness traits. This observation aligns with the broad roles of lipids in viral entry and replication (Heaton and Randall, 2011) and the requirement of *sbp-1* for OrV replication (Casorla-Perez et al., 2022).

### 3.3 RSEs and LSMMG attenuate canonical infection responses

**Figure 2:**
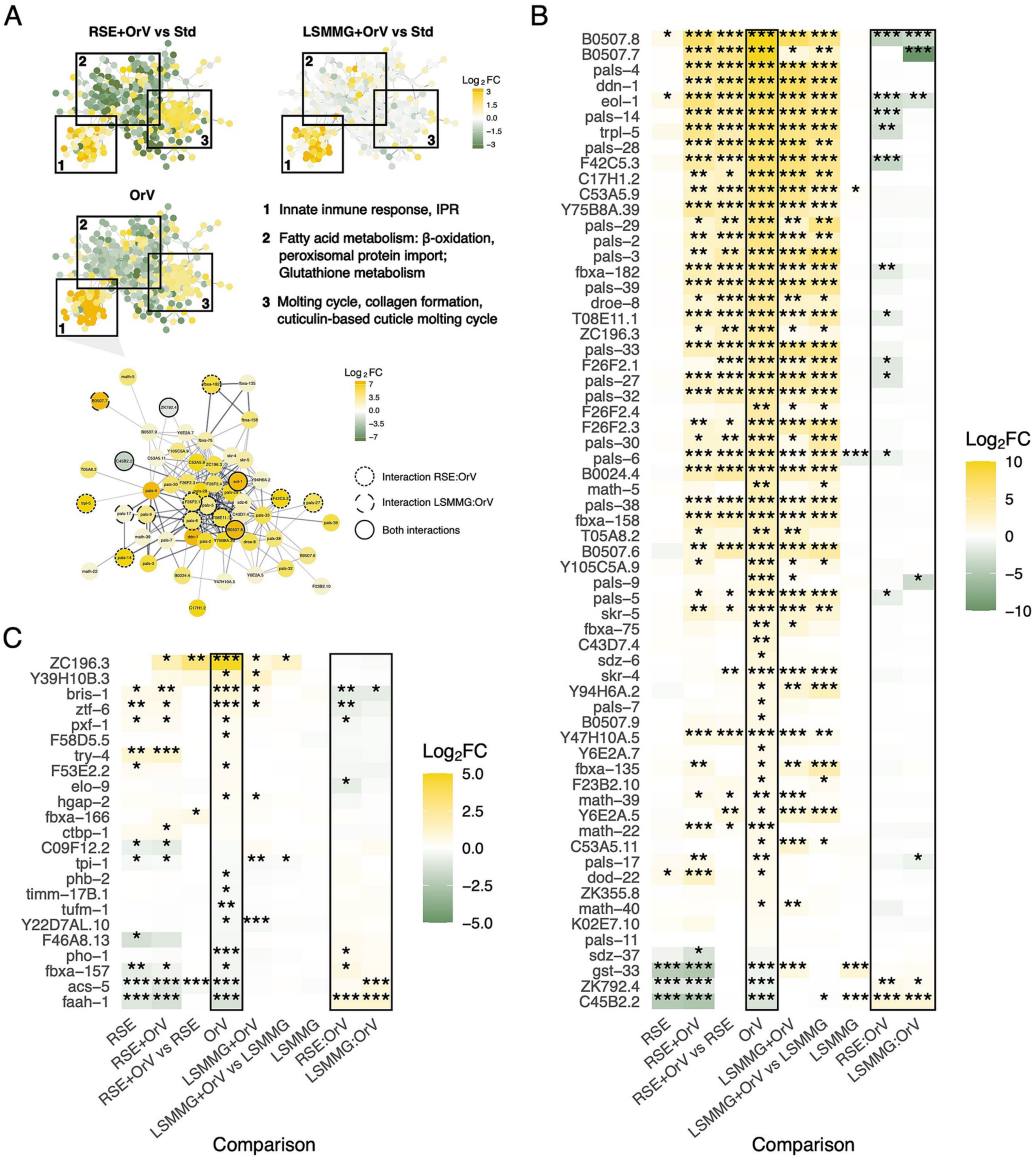
**(A)** Cluster from a STRING-based network of genes with ∣log_2_*FC*∣ > 1 in response to OrV infection, including infection-specific response genes. The three main gene communities are highlighted with squares, and their predominant biological processes are indicated. In the community of infection-specific response genes, log_2_*FC* values are shown for both RSE + OrV *vs.* standard (Std) and LSMMG + OrV *vs.* Std comparisons. In the community of infection-specific response genes, circle color also represents log₂*FC* values, while the outline style denotes genes showing significant interaction effects between RSEs or LSMMG and OrV infection. Asterisks denote adjusted significance levels (^*^p < 0.05, ^**^p < 0.01, ^***^p < 0.001). **(B)** Genes from **(A)** show log_2_*FC* across different conditions (relative to non-infected nematodes in each condition, unless otherwise indicated) and for the interactions RSE × OrV and LSMMG × OrV. **(C)** Genes previously identified as correlating or anti-correlating with OrV accumulation, displaying log_2_*FC* across conditions and for the interactions RSE × OrV and LSMMG × OrV.

STRING-based clustering of infection-responsive genes uncovered modules whose amplitude depended on the physical background, showing stronger differences in the LSMMG + OrV network (Figure 2A). To assess whether antagonistic interactions affect specific infection pathways, we examined the expression of previously characterized OrV infection-responsive genes (Castiglioni et al., 2024), whose cluster exhibited the most robust expression (Figures 2A,B). Under RSEs alone, *B0507.8*, *dod-22,* and *eol-1* were mildly increased, while *C45B2.2*, *gst-33,* and *ZK792.4* were reduced. In RSE + OrV, 11 genes—including IPR/innate immunity effectors *pals-5*, *pals-6*, *pals-14*, *pals-27*, and *F26F2.1*—showed weaker upregulation than under OrV alone (Huang et al., 2021; Castiglioni et al., 2024; Lažetić et al., 2022). The infection-inducible *eol-1* (Shen et al., 2014) and *trpl-5* (Goodman, 2006) were also attenuated, indicating partial priming or antagonism. Conversely, the downregulation of *ZK792.4* and *C45B2.2* under RSEs was not significantly affected by OrV, maintaining RSE-baseline levels.

For LSMMG + OrV, five IPR components (*B0507.7*, *B0507.8*, *eol-1*, *pals-9*, and *pals-17*) displayed lower expression than expected from the additive effects of the single stresses (Lažetić et al., 2022; Lažetić et al., 2023). Load-correlated infection markers (*bris-1*, *elo-9*, *pxf-1*, and *ztf-6*) were buffered by LSMMG and RSEs, and both stresses interfered with the canonical downregulation of *faah-1* (Nandakumar and Tan, 2008; Harrison et al., 2014; Ruiz et al., 2019) (Figure 2C). Together, these trends support a stress-primed, antagonistic interaction: The environmental background tempers hallmark infection programs while preserving selective defense and metabolic adjustments.

### 3.4 Stress-specific transcriptional signatures reveal distinct modulation of the viral infection response

**Figure 3:**
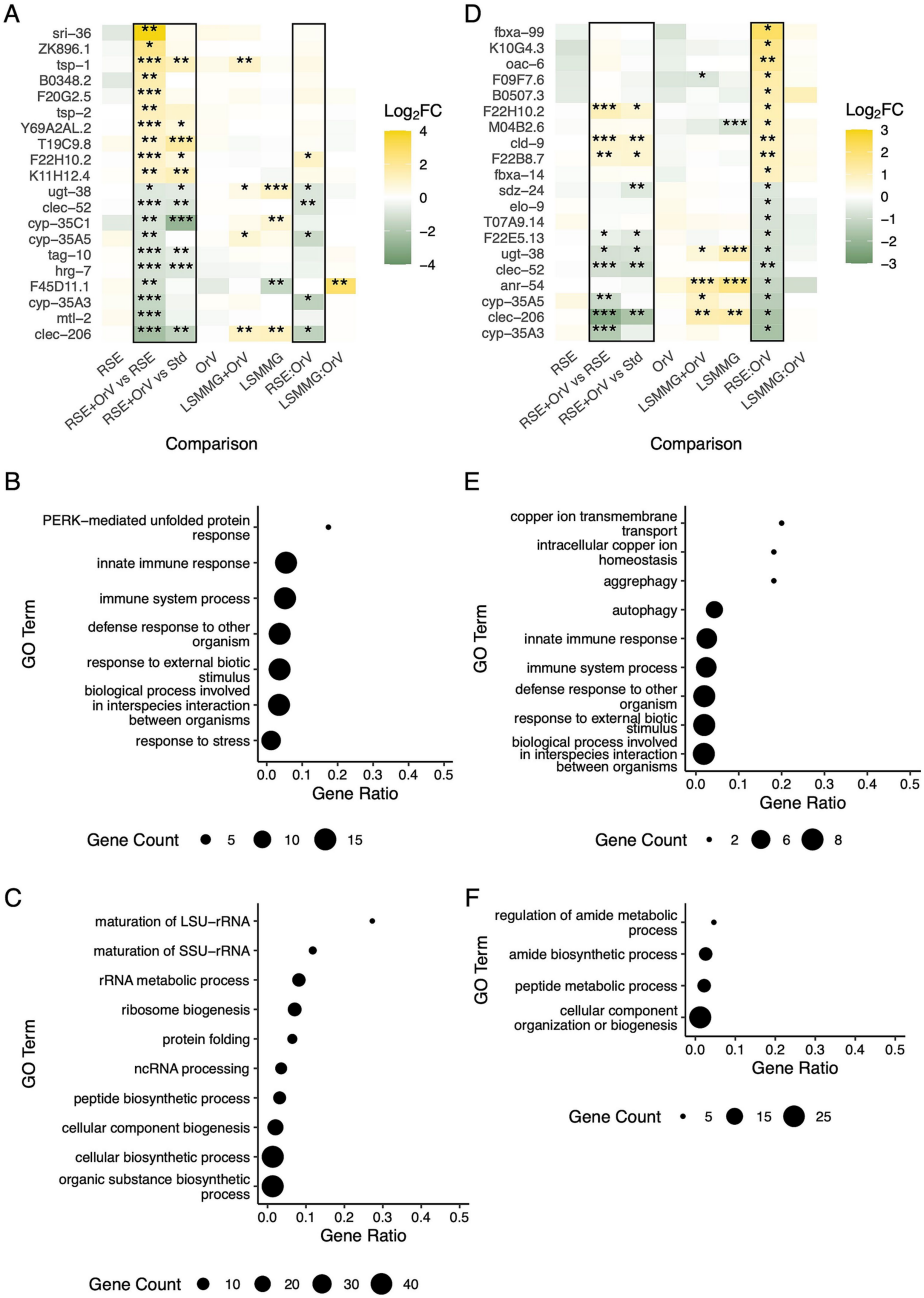
**(A)** Panel of the most significantly upregulated or downregulated genes exclusively under RSE conditions after OrV inoculation (14 hpi), compared to non-infected nematodes in this condition. Log_2_*FC* values across all analyzed conditions and interaction effects are shown. Unless otherwise stated, all comparisons were made relative to non-infected nematodes maintained under standard conditions (Std). Asterisks indicate adjusted significance levels (^*^p < 0.05, ^**^p < 0.01, ^***^p < 0.001). **(B)** GO enrichment analysis of the top biological processes based on exclusively and significantly (adjusted *p* < 0.01) upregulated genes in the comparison shown in **(A)**. **(C)** GO enrichment analysis of the top biological processes based on exclusively and significantly (adjusted *p* < 0.01) downregulated genes in the comparison shown in **(A)**. **(D)** Panel of the 10 most significantly and exclusively upregulated and downregulated genes for the RSE × OrV interaction, displayed as in **(A)**. **(E)** GO enrichment analysis of the top biological processes based on exclusively and significantly upregulated genes for this interaction. **(F)** GO enrichment analysis of the top biological processes based on exclusively and significantly downregulated genes for this interaction, presented as in **(B)**.

Beyond shared antagonism, RSEs and LSMMG produced stress-specific transcriptional signatures. Under RSE + OrV (*vs.* RSEs), we detected 233 upregulated and 197 downregulated genes, largely overlapping with the standard infection signature. Excluding genes shared with individual stresses yielded 89 upregulated and 94 downregulated genes, which were further reduced to 15 uniquely regulated genes after filtering for |log_2_*FC*| > 1 (Figure 3A). These included *sri-36* (serpentine receptor), tetraspanins *tsp-1* and *tsp-2*, and ion-binding proteins such as *Y69A2AL.2* (phospholipid-binding prediction), consistent with barrier remodeling and durable stress memory (Jiang et al., 2024). Conversely, *mtl-2* and several cytochrome P450s (e.g., *cyp-35A3, cyp-35A5,* and *cyp-35C1*) were decreased, alongside *clec-206*, suggesting deliberate restraint of detoxification to limit reactive intermediates (Hall et al., 2012). GO terms enriched among upregulated genes included immune defense and, notably, the predominant PERK-mediated UPR (Figure 3B) (Castiglioni et al., 2024; Kim et al., 2020; Brown et al., 2021; Zheng et al., 2022; Son et al., 2024), reinforcing that RSEs acts as a physiological stressor and aligning with hormesis-like responses. GO terms overrepresented in downregulated genes included rRNA metabolism and ribosome biogenesis, consistent with proteostasis challenges (Figure 3C).

Among genes showing a significant interaction between RSEs and infection status, we identified 1,940 genes. Among those with the strongest interaction, we found *argk-1* (creatine kinase linked to stress resistance and lifespan), *col-147* (collagen), *fipr-7* (feeding), *ttr-21*, and *Y73F4.2* (pathogen response) (McQuary et al., 2016; Burton et al., 2021; Sheshadri et al., 2021; Li et al., 2025). In contrast, infection-inducible genes *B0507.8*, *eol-1*, and *F42C5.3* were attenuated (higher than under RSEs alone but lower than under OrV alone) (Castiglioni et al., 2024; Teuscher et al., 2019; Van Sluijs et al., 2022) (Supplementary Figure S3A). GO analysis of |log_2_*FC*| > 1 genes showed supra-additive enrichment for lipid catabolism, detoxification, and immunity among interaction-up genes (383), while interaction-down genes (262) were enriched for cuticle development, genitalia and sensory development, and protein auto-processing (Supplementary Figure S3B). Collagen modules, linked to RSEs and antiviral activity, were prominent (Van Voorhies et al., 2020; Zhou et al., 2024), consistent with prior observations of altered reproduction under RSE + OrV relative to RSEs alone (Villena-Giménez et al., 2025). Excluding significant genes shared with RSEs or OrV effects, 59 and 66 genes were identified as interaction-specific induced and repressed, respectively (Figure 3D). GO enrichment analysis revealed that the exclusively upregulated genes were associated with copper homeostasis, autophagy, and immune system processes (Figure 3E), while the downregulated genes were enriched for amide biosynthesis and peptide metabolism under combined stress conditions (Figure 3F).

**Figure 4:**
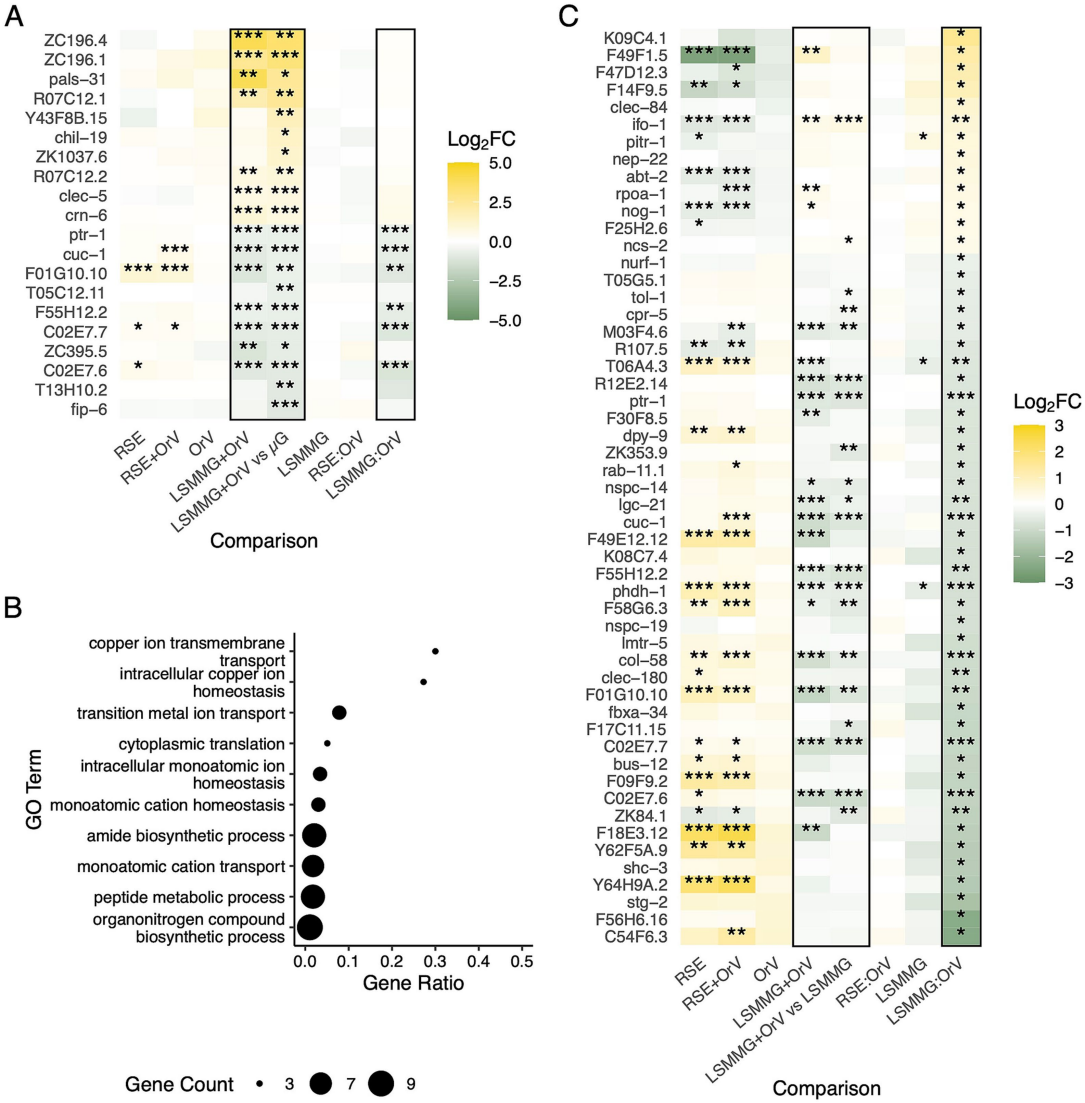
**(A)** Panel of the most significantly and exclusively upregulated or downregulated genes under LSMMG conditions after OrV inoculation (14 hpi), compared to non-infected nematodes in the same condition. Log_2_*FC* values across all analyzed conditions and interaction effects are shown. Unless otherwise stated, all comparisons were made relative to non-infected nematodes maintained under standard conditions (Std). Asterisks indicate adjusted significance levels (^*^p < 0.05, ^**^p < 0.01, ^***^p < 0.001). **(B)** GO enrichment analysis of the top biological processes based on exclusively and significantly (adjusted *p* < 0.01) downregulated genes in the comparison shown in **(A)**. **(C)** Panel of exclusively and significantly upregulated and downregulated genes for the LSMMG × OrV interaction, displayed as in **(A)**.

OrV infection under microgravity (LSMMG + OrV *vs.* LSMMG) produced fewer unique genes, with eight exhibiting ∣log₂*FC*∣ > 1. Of these, 48 upregulated and 71 downregulated genes were absent from the single-stress lists, including *pals-31* and *chil-19* (carbohydrate metabolism) (Figure 4A). GO terms among the downregulated genes pointed to amide biosynthesis, peptide metabolism, and metal transport and homeostasis (particularly copper), consistent with LSMMG-linked bone phenotypes and copper’s multifaceted role in immunity and viral life cycles (Man et al., 2022; Albalawi et al., 2024) (Figure 4B). Several strongly regulated single-stress genes reverted toward baseline in the combination (Supplementary Figure S3C): *fipr-4, fipr-7*, and *fipr-10* (defense and cuticle) (Kamal et al., 2022); *grd-17* (hedgehog-like signaling implicated in host–microbe interactions) (Zárate-Potes et al., 2022); and IPR components *B0507.7* and *B0507.8*. Using |log_2_*FC*| > 1, overexpressed genes showing a significant interaction between gravity intensity and infection status were enriched for sphingomyelin biosynthesis, medium-chain fatty acid catabolism, and cellular detoxification. Interaction-down genes emphasized cuticle and collagen, an LSMMG hallmark (Çelen et al., 2023), although this is complicated by the antiviral roles of collagens in OrV (Zhou et al., 2019) (Supplementary Figure S3D).

After removing genes significantly involved in this interaction, 13 upregulated and 40 downregulated genes remained (Figure 4C), with no significant enrichment in any biological process. Most of these exclusive genes exhibited a suppressive combined response that emerged only under dual exposure. The most strongly suppressed gene, *C54F6.3*, a chondroitin 4-sulfotransferase (Mizuguchi et al., 2009), along with collagens and C-type lectins [GO:0005615 (extracellular space), odds ratio = 11.262, *p* = 0.017], suggests that the stress combination heightens cellular vulnerability to oxidative or inflammatory damage, weakening extracellular matrix-mediated protection. Together with the reduced overall DEGs under LSMMG + OrV, these data reinforce the notion of transcriptional attenuation and resource conservation under multi-stress exposure.

### 3.5 Viral genomic features under stress: replication state, sequence diversity, and nsVG architecture

**Figure 5:**
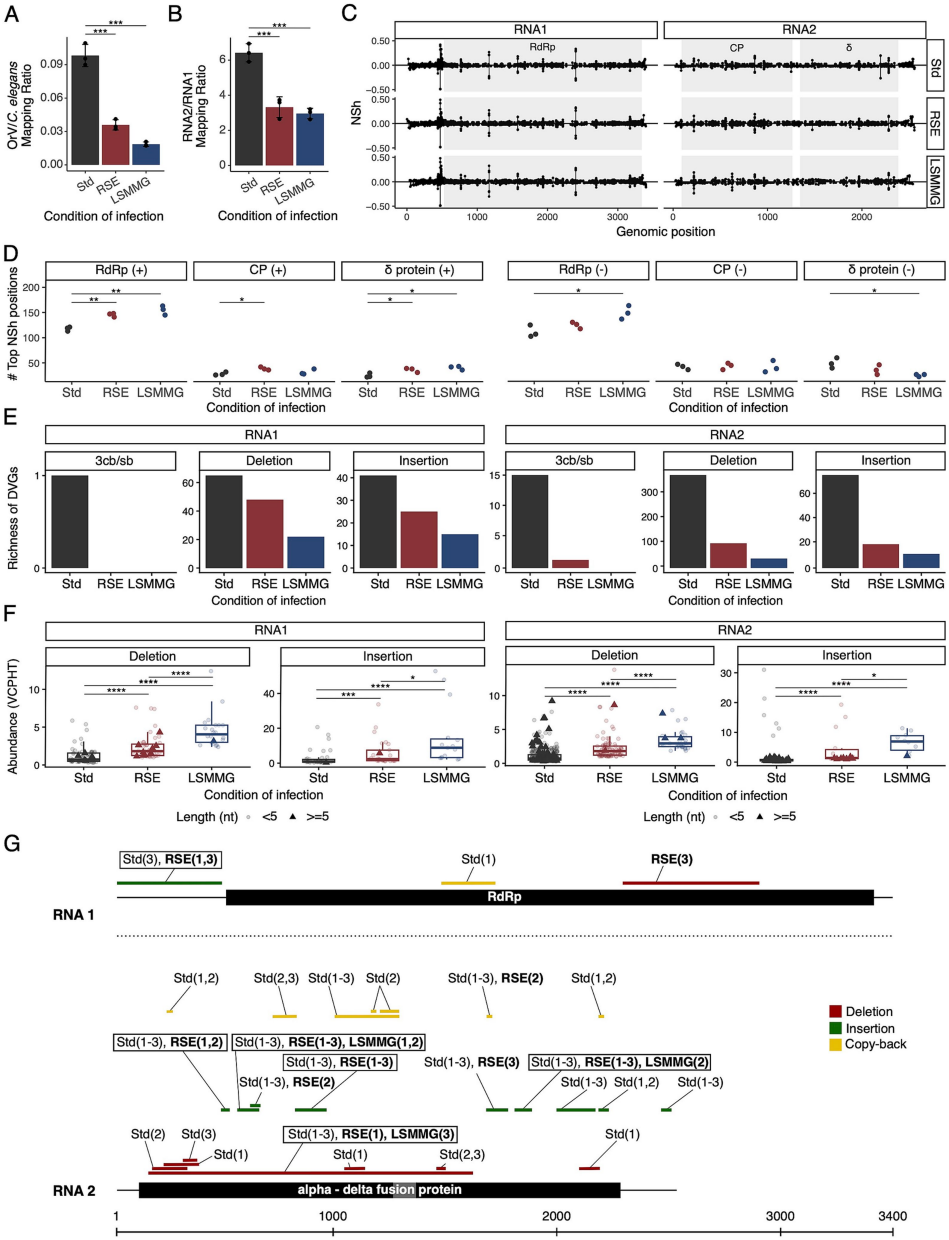
**(A)** Bar plot shows OrV-to-*C. elegans* mapping ratios across infection conditions, presented as mean ±1 SD. The ratios significantly differed among the three experimental conditions (Welch robust ANOVA: *F*_2,3.145_ = 85.794, *p* = 0.002, η^2^ = 0.977), with the differences attributable to the reduction of the ratio under the two stress conditions compared to standard conditions (Bonferroni *post hoc* test, *p* < 0.001). **(B)** Bar plot illustrates RNA2/RNA1 mapping ratios across the three infection conditions, with mean ±1 SD. The ratios significantly differed among the three experimental conditions (Welch robust ANOVA: *F*_2,3.638_ = 43.476, *p* = 0.003, η^2^ = 0.939), with the differences attributable to the reduction of the ratio under the two stress conditions compared to standard conditions (Bonferroni *post hoc* test, *p* < 0.001). **(C)** Normalized Shannon entropy (*H_n_*) values along viral genome sequences; coding sequences (CDSs) are indicated by shaded regions. **(D)** Number of genomic positions within the top 5% most variable sites, with *H_n_* values shown for the positive and negative strands. Statistical significance was assessed using a *t*-test; asterisks indicate significance (^*^p < 0.05, ^**^p < 0.01). **(E)** Richness of non-standard viral genomes (nsVGs), including copy-backs, insertions, and deletions, detected using the DVGfinder metasearch algorithm (option ViReMa). **(F)** Abundance of nsVGs is expressed as counts per 100,000 viral reads (VCPHT), detected using DVGfinder (with ViReMa). Statistical significance was evaluated using a Wilcoxon test; asterisks denote significance (^*^p < 0.05, ^**^p < 0.01, ^***^p < 0.001, ^****^p < 0.0001). **(G)** Consensus nsVGs detected across samples using DVGfinder in consensus mode, indicating the specific samples in which the events were identified. nsVGs are color-coded by type, and boxed regions highlight those detected in at least five samples and under two or more conditions.

To assess whether transcriptional antagonism translated into viral fitness consequences, we quantified viral RNA accumulation (viral load) and characterized viral population genomes. Viral load (quantified based on viral reads from the RNA-seq data) decreased under RSEs and dropped further under LSMMG. This decline was driven largely by RNA2, reducing the RNA2/RNA1 ratio and suggesting that infections were at an earlier stage of the replication program despite identical sampling windows (Figures 5A,B) (Castiglioni et al., 2024).

Normalized Shannon entropy profiles were broadly similar across conditions, but the number of high-entropy positions (top 5%) shifted by strand and coding region (Figures 5C,D). On the positive strand, high-entropy sites increased under RSEs and LSMMG within RdRp and *δ* (and CP under RSEs); on the negative strand, RdRp increased under LSMMG, although the overall number of high-entropy sites decreased under LSMMG. Given that RdRp precedes δ and CP in expression (Castiglioni et al., 2024), these data support a replication-timing mechanism modulated by environmental stress. Mechanistically, elevated ROS in stressed hosts may influence viral diversity by introducing RNA lesions that perturb polymerase extension (Akaike and Maeda, 2000; Akagawa et al., 2025), while certain RNA viruses can exploit oxidative cues to fine-tune capping and replication timing (Gullberg et al., 2015).

Non-standard viral genomes (nsVGs) added a structural dimension. Under stringent filtering, richness tracked load (Figure 5E). However, after normalizing by viral reads (VCPHT), insertions and deletions were more abundant under RSEs and further increased under LSMMG (Figure 5F), indicating greater structural variation per unit of viral RNA when replication is constrained. High-confidence, conserved nsVGs included duplications in RNA1 (5’-UTR, capsid) and a recurrent RNA2 deletion spanning CP into δ (Figure 5G), highlighting CP as a hotspot for quasispecies diversification. An RSE-specific > 5-nt RNA1 deletion (absent in other conditions despite higher standard condition loads) reached up to 6.95 VCPHT, consistent with an RSE muon flux host environment that stabilizes specific nsVGs or transient defective RNA1 compositions.

### 3.6 A working model for non-additive stress integration

Across datasets, we observed (*i*) acclimation, (*ii*) antagonism between abiotic stress and infection responses, and (*iii*) proteostasis- and resource-centric reprogramming. These patterns converge on a model in which chronic environmental stress primes transcriptional circuits that interfere with acute infection responses. We propose that multi-stress exposure activates compensatory circuits (e.g., IPR modulation, PERK/ER-stress signaling, redox management, and lipid rerouting) that attenuate hallmark infection cascades while maintaining essential defenses and metabolic balance (Reddy et al., 2017; Kim et al., 2020; Brown et al., 2021; Zheng et al., 2022; Son et al., 2024). The repeated involvement of lipid and collagen/cuticle modules points to barrier function and membrane remodeling as key mechanisms through which space-analog stress influences both host resilience and viral replication (Çelen et al., 2023; Van Voorhies et al., 2020; Zhou et al., 2024).

### 3.7 Limitations of the study

The combined LSMMG + RSE + OrV condition could not be assayed due to logistical constraints. The complex logistics of these experiments limited the feasibility of testing multiple genetic backgrounds and time points. Genetic dissection using mutant strains (e.g., *sbp-1* and *mdt-15*), time-resolved multi-omics (i.e., transcriptome, proteome, and lipidome), and longitudinal nsVG tracking to test causality remain priorities for future research. Mapping conserved modules (e.g., PERK signaling, copper homeostasis, and collagen/extracellular matrix) in mammalian systems will be essential for anticipating immune modulation and viral reactivation risk during space exploration missions (Tanaka et al., 2017; Wu et al., 2024; Mehta et al., 2017; Restier-Verlet et al., 2021; Atri and Melott, 2011; Atri and Melott, 2014) and for designing effective countermeasures.

## 4 Conclusion

Acclimation to simulated microgravity and reduced muon flux dampens the canonical OrV infection program and reconfigures host physiology, yielding antagonistic (non-additive) outcomes across gene modules and viral phenotypes. On the host side, we observed selective attenuation of IPR modules, redistribution of lipid and metal (copper) homeostasis, and recurrent impacts on collagen/cuticle biology. On the viral side, we found reduced load, lower RNA2/RNA1 ratios, strand- and CDS-specific entropy shifts, and increased per-read structural variation, with the capsid region emerging as a mutational hotspot. These findings support the idea that stress-primed transcriptional attenuation and resource allocation act as strategies to limit proteotoxic burden under multi-stress exposure while reshaping replication timing and genome plasticity.

## Data Availability

The datasets presented in this study can be found in online repositories. RNA-seq data were deposited at the European Nucleotide Archive (ENA) at EMBL-EBI under accession number PRJEB102402 (https://www.ebi.ac.uk/ena/browser/view/PRJEB102402). All the R code generated for this study are accessible in Zenodo at https://doi.org/10.5281/zenodo.17607908.

## Footnotes

1
https://www.endmemo.com/bio/dnacopynum.php

2
www.novogene.com
