# Supplementary material for: Cosmic silence and viral noise: transcriptomic crosstalk in Caenorhabditis elegans under simulated space conditions
Source: Front Microbiol. 2026 Mar 18;17:1781245. doi: 10.3389/fmicb.2026.1781245 (PMC13039024; doi:10.3389/fmicb.2026.1781245)
Supplement: Supplementary file 1 [file Supplementary_file_1.docx]

**Supplementary Materials**

**Cosmic silence and viral noise: transcriptomic crosstalk in *Caenorhabditis elegans* under simulated space conditions**

**Ana Villena-Giménez^1,*^, Esmeralda G. Legarda^1,*^, Rubén González^2^ Victoria G. Castiglioni^1,3^, and Santiago F. Elena^1,4^**

Figures S1 – S3.

Tables S1 – S3.

**Figure S1.** General overview of differential gene expression across samples and conditions. (A) Principal component analysis (PCA) of the samples based on gene expression profiles. after a variance stabilizing transformation (VST). Scores are shown for the first and third principal components (PC1 and PC3). which together explain 85% of the total variance. Each point represents an individual sample. shaped by abiotic condition (standard (Std), radiologically shielded environments (RSE) or low shear modeled microgravity (LSMMG)) and colored according to infection status by OrV. Separation along PC1 reflects abiotic-stress conditions. while variation along PC3 captures infection. (B) Volcano plot showing differential expression results for OrV infection. The *x*-axis represents the log_2_*FC*. and the *y*-axis represents the –log_10_ ajusted *P*. Significantly expressed genes are highlighted in blue (light blue: |log_2_*FC*| ≤ 2; dark blue: |log_2_*FC*| > 2). Genes with highest expression are labeled. (C) Volcano plot showing differential expression results for RSE. displayed as in (B). (D) Volcano plot showing differential expression results for LSMMG. displayed as in (B). (E) Volcano plot showing differential expression results for infected samples under RSE *vs* non-infected samples in standard conditions (RSE + OrV *vs* Std). displayed as in (B). (F) Volcano plot showing differential expression results for infected samples under LSMMG *vs* non-infected samples in standard conditions (LSMMG + OrV *vs* Std). displayed as in (B). (G) Volcano plot showing differential expression results for OrV infection in RSE (RSE + OrV *vs* RSE). displayed as in (B). (H) Volcano plot showing differential expression results for OrV infection in LSMMG (LSMMG + OrV *vs* LSMMG).

**Figure S2**. (A) Panel of upregulated core response genes to OrV infection (14 hpi) for three different conditions (standard (Std). radiologically shielded environments (RSE) or microgravity (LSMMG)) *vs* its corresponding basal condition. Log_2_*FC* values across all analyzed conditions and interaction effects are shown. Unless otherwise stated. all comparisons shown were made relative to non-infected nematodes maintained under standard conditions (Std). Asterisks indicate adjusted significance levels (^*^*P* < 0.05. ^**^*P* < 0.01. ^***^*P* < 0.001). (B) Panel of downregulated core response genes to OrV infection for three different conditions vs its corresponding basal condition. displayed as in (A). (C) GO enrichment analysis of biological processes based on upregulated (up) and downregulated (down) core response genes (terms with adjusted P < 0.01) shown in (A) and (B). (D) Venn diagram showing intersection between significant genes for non-additive effects of RSE and OrV (RSE × OrV). with those for LSMMG and OrV interaction (LSMMG × OrV). (E) Panel of proviral genes described for OrV to date. displayed as in (A). (F) Panel of fatty-acid regulators.

**Figure S3.** (A) Panel of the ten most significantly upregulated and downregulated genes for the radiologically shielded environments (RSE) × OrV interaction. Log_2_*FC* values across all analyzed conditions and interaction effects are shown. Unless otherwise stated. all comparisons were made relative to non-infected nematodes maintained under standard conditions (Std). Asterisks indicate adjusted significance levels (^*^P < 0.05. ^**^P < 0.01. ^***^P < 0.001). (B) GO enrichment analysis of top biological processes based on all significantly expressed genes for the interaction RSE × OrV with a |log_2_*FC*| > 1. Categories include global upregulation (left) and downregulation (right). with all adjusted P < 0.01. (C) Panel of the twenty most significantly upregulated and downregulated genes for the microgravity (LSMMG) × OrV interaction. displayed as in (A). (D) GO enrichment analysis of top biological processes based on all significantly expressed genes with |log_2_*FC*| > 1 for the interaction LSMMG × OrV.

| **Table S1.** List of primers used for OrV quantification | | |  |
| --- | --- | --- | --- |
| **Primer name** | **Sequence** | **Application** | |
| oVG7_OrV_RNA2_3'_F | TAATACGACTCACTATAGGCCTGTCAGAGTTGAGAACA | Standard curve | |
| oVG8_OrV_RNA2_3'_R | ATAGCCGGGTATGGATAGCG | Standard curve | |
| oVG15_RNA2_qPCR_2_F | ACGAAGCAGTAGCCGTTAAG | qRT-PCR OrV | |
| oVG16_RNA2_qPCR_2_R | GAGAACATCCTTCTCTGCGG | qRT-PCR OrV | |

| **Table S2**. Core response genes to OrV across conditions, showing adjusted *P* and log_2_*FC* for each condition or comparison. | | | |
| --- | --- | --- | --- |
| **Gene** | **log_2_ Fold change** | **Adjusted *P*** | **Condition/Comparison** |
| *B0024.4* | -0.2615 | 0.2733 | RSE |
| *pals-27* | 0.3426 | 0.4124 | RSE |
| *pals-28* | 0.0734 | 1.0000 | RSE |
| *valv-1* | -0.0647 | 1.0000 | RSE |
| *ddn-1* | -0.0737 | 0.7400 | RSE |
| *B0507.6* | -0.7942 | 0.1066 | RSE |
| *B0507.7* | 0.1287 | 1.0000 | RSE |
| *B0507.8* | 1.1536 | 0.0258 | RSE |
| *C06E4.8* | -0.0384 | 1.0000 | RSE |
| *fbxa-158* | -0.0165 | 1.0000 | RSE |
| *math-14* | -0.4671 | 0.4670 | RSE |
| *C17H1.2* | 0.1088 | 1.0000 | RSE |
| *pals-2* | 0.2120 | 1.0000 | RSE |
| *pals-3* | -0.0681 | 1.0000 | RSE |
| *pals-4* | 0.2084 | 0.6937 | RSE |
| *pals-5* | 0.1137 | 1.0000 | RSE |
| *pals-6* | -0.0091 | 1.0000 | RSE |
| *ppw-1* | 0.3550 | 0.0176 | RSE |
| *C18H7.11* | 0.2082 | 0.5273 | RSE |
| *C27D9.2* | 1.1236 | 0.0024 | RSE |
| *rcs-1* | 0.4240 | 0.0063 | RSE |
| *pals-32* | -0.1744 | 0.7795 | RSE |
| *C49C3.9* | 0.2068 | 0.2938 | RSE |
| *C49C8.8* | 0.0175 | 1.0000 | RSE |
| *C53A5.9* | 0.1299 | 0.9847 | RSE |
| *pals-36* | -0.4746 | 0.4920 | RSE |
| *pals-37* | -0.0647 | 1.0000 | RSE |
| *pals-39* | -0.0649 | 0.7284 | RSE |
| *pals-38* | -0.0058 | 1.0000 | RSE |
| *clec-45* | 0.4144 | 0.3410 | RSE |
| *F07E5.9* | -0.0678 | 1.0000 | RSE |
| *F15H10.10* | 0.0690 | 1.0000 | RSE |
| *F15H10.5* | -0.3392 | 0.5931 | RSE |
| *pals-14* | 0.2254 | 0.3392 | RSE |
| *F26D11.13* | -0.0634 | 1.0000 | RSE |
| *F26F2.1* | -0.1099 | 1.0000 | RSE |
| *F26F2.3* | 0.0996 | 1.0000 | RSE |
| *F35E12.10* | -0.2175 | 0.2217 | RSE |
| *F42C5.3* | 0.6589 | 0.1760 | RSE |
| *fbxa-182* | 0.2458 | 0.3102 | RSE |
| *skr-5* | 0.2623 | 0.9326 | RSE |
| *fbxa-188* | 0.3637 | 0.6042 | RSE |
| *sago-2* | 0.4391 | 0.0136 | RSE |
| *F57G4.11* | -0.1223 | 1.0000 | RSE |
| *K10G4.13* | 0.0649 | 1.0000 | RSE |
| *M01G12.7* | -0.2533 | 1.0000 | RSE |
| *lec-8* | -0.2567 | 0.1752 | RSE |
| *T07H8.11* | 0.3881 | 0.6643 | RSE |
| *T08E11.1* | 0.1130 | 1.0000 | RSE |
| *pho-14* | -0.1642 | 0.3193 | RSE |
| *trpl-5* | 1.0290 | 0.0689 | RSE |
| *T19D12.4* | -0.0715 | 0.6918 | RSE |
| *eol-1* | 1.2415 | 0.0109 | RSE |
| *pals-29* | 0.1074 | 1.0000 | RSE |
| *pals-33* | 0.1077 | 1.0000 | RSE |
| *Y105C5A.1269* | -0.0261 | 1.0000 | RSE |
| *lys-1* | 0.1475 | 0.2262 | RSE |
| *lys-3* | 0.1665 | 0.3878 | RSE |
| *Y46G5A.20* | -0.1532 | 0.4666 | RSE |
| *Y47H10A.5* | 0.0253 | 0.7965 | RSE |
| *pals-30* | 0.2367 | 1.0000 | RSE |
| *skr-4* | -0.0694 | 1.0000 | RSE |
| *Y6E2A.5* | -0.2226 | 1.0000 | RSE |
| *Y75B8A.39* | 0.4331 | 0.5871 | RSE |
| *ZC196.3* | 0.0406 | 1.0000 | RSE |
| *droe-8* | 0.1430 | 1.0000 | RSE |
| *ZK896.4* | 0.0807 | 1.0000 | RSE |
| *B0024.4* | 0.0557 | 0.6651 | LSMMG |
| *pals-27* | 0.1081 | 0.4610 | LSMMG |
| *pals-28* | 0.0471 | 0.7091 | LSMMG |
| *valv-1* | 0.0007 | 1.0000 | LSMMG |
| *ddn-1* | 0.1320 | 0.2107 | LSMMG |
| *B0507.6* | -0.1718 | 0.2230 | LSMMG |
| *B0507.7* | 0.0585 | 0.5801 | LSMMG |
| *B0507.8* | 0.3020 | 0.0811 | LSMMG |
| *C06E4.8* | -0.0234 | 1.0000 | LSMMG |
| *fbxa-158* | -0.0749 | 0.6577 | LSMMG |
| *math-14* | -0.0088 | 1.0000 | LSMMG |
| *C17H1.2* | 0.0229 | 1.0000 | LSMMG |
| *pals-2* | 0.0605 | 0.7256 | LSMMG |
| *pals-3* | -0.0264 | 0.9666 | LSMMG |
| *pals-4* | 0.0635 | 0.5395 | LSMMG |
| *pals-5* | -0.0124 | 1.0000 | LSMMG |
| *pals-6* | -1.4165 | 0.0001 | LSMMG |
| *ppw-1* | 0.1384 | 0.1352 | LSMMG |
| *C18H7.11* | 0.0465 | 0.7197 | LSMMG |
| *C27D9.2* | -0.0299 | 0.8215 | LSMMG |
| *rcs-1* | -0.0524 | 0.6022 | LSMMG |
| *pals-32* | -0.0715 | 0.5571 | LSMMG |
| *C49C3.9* | 0.4969 | 0.0039 | LSMMG |
| *C49C8.8* | 0.4989 | 0.0490 | LSMMG |
| *C53A5.9* | 0.4416 | 0.0488 | LSMMG |
| *pals-36* | -0.0951 | 0.5622 | LSMMG |
| *pals-37* | -0.3670 | 0.0611 | LSMMG |
| *pals-39* | 0.0007 | 1.0000 | LSMMG |
| *pals-38* | -0.0461 | 0.9066 | LSMMG |
| *clec-45* | -0.0473 | 1.0000 | LSMMG |
| *F07E5.9* | -0.1104 | 0.3377 | LSMMG |
| *F15H10.10* | 0.0012 | 1.0000 | LSMMG |
| *F15H10.5* | -0.0465 | 1.0000 | LSMMG |
| *pals-14* | 0.0324 | 0.7759 | LSMMG |
| *F26D11.13* | 0.0475 | 1.0000 | LSMMG |
| *F26F2.1* | -0.1918 | 0.1789 | LSMMG |
| *F26F2.3* | -0.1090 | 0.4800 | LSMMG |
| *F35E12.10* | 0.5625 | 0.0000 | LSMMG |
| *F42C5.3* | 0.0618 | 0.6574 | LSMMG |
| *fbxa-182* | -0.1512 | 0.2762 | LSMMG |
| *skr-5* | 0.0366 | 0.9824 | LSMMG |
| *fbxa-188* | -0.0711 | 0.7439 | LSMMG |
| *sago-2* | 0.1506 | 0.1574 | LSMMG |
| *F57G4.11* | -0.0202 | 0.9239 | LSMMG |
| *K10G4.13* | -0.0016 | 1.0000 | LSMMG |
| *M01G12.7* | -0.0309 | 0.9543 | LSMMG |
| *lec-8* | -0.0496 | 0.6782 | LSMMG |
| *T07H8.11* | 0.0615 | 0.7614 | LSMMG |
| *T08E11.1* | -0.1770 | 0.2230 | LSMMG |
| *pho-14* | -0.0274 | 0.8120 | LSMMG |
| *trpl-5* | 0.0083 | 1.0000 | LSMMG |
| *T19D12.4* | -0.1075 | 0.3627 | LSMMG |
| *eol-1* | 0.2608 | 0.1120 | LSMMG |
| *pals-29* | -0.0196 | 1.0000 | LSMMG |
| *pals-33* | 0.0152 | 0.8813 | LSMMG |
| *Y105C5A.1269* | 0.0652 | 0.9290 | LSMMG |
| *lys-1* | -0.0499 | 0.5312 | LSMMG |
| *lys-3* | 0.1277 | 0.3364 | LSMMG |
| *Y46G5A.20* | -0.0609 | 0.6414 | LSMMG |
| *Y47H10A.5* | 0.1035 | 0.4245 | LSMMG |
| *pals-30* | -0.0515 | 1.0000 | LSMMG |
| *skr-4* | 0.0999 | 0.4396 | LSMMG |
| *Y6E2A.5* | 0.0085 | 1.0000 | LSMMG |
| *Y75B8A.39* | 0.1287 | 0.4045 | LSMMG |
| *ZC196.3* | 0.0109 | 1.0000 | LSMMG |
| *droe-8* | 0.0338 | 1.0000 | LSMMG |
| *ZK896.4* | -0.1476 | 0.2815 | LSMMG |
| *B0024.4* | 3.7345 | 0.0000 | OrV |
| *pals-27* | 4.3391 | 0.0000 | OrV |
| *pals-28* | 6.7640 | 0.0000 | OrV |
| *valv-1* | 2.2830 | 0.0000 | OrV |
| *ddn-1* | 7.7734 | 0.0000 | OrV |
| *B0507.6* | 3.1875 | 0.0000 | OrV |
| *B0507.7* | 9.5991 | 0.0000 | OrV |
| *B0507.8* | 9.7142 | 0.0000 | OrV |
| *C06E4.8* | 2.5970 | 0.0007 | OrV |
| *fbxa-158* | 3.5565 | 0.0000 | OrV |
| *math-14* | 2.5381 | 0.0077 | OrV |
| *C17H1.2* | 6.5293 | 0.0002 | OrV |
| *pals-2* | 5.7829 | 0.0000 | OrV |
| *pals-3* | 5.3409 | 0.0000 | OrV |
| *pals-4* | 8.4802 | 0.0000 | OrV |
| *pals-5* | 3.0103 | 0.0000 | OrV |
| *pals-6* | 3.8455 | 0.0000 | OrV |
| *ppw-1* | 2.1952 | 0.0000 | OrV |
| *C18H7.11* | 1.0698 | 0.0025 | OrV |
| *C27D9.2* | 1.4714 | 0.0000 | OrV |
| *rcs-1* | 0.7323 | 0.0000 | OrV |
| *pals-32* | 4.2372 | 0.0000 | OrV |
| *C49C3.9* | 0.9554 | 0.0006 | OrV |
| *C49C8.8* | 3.3853 | 0.0000 | OrV |
| *C53A5.9* | 6.2245 | 0.0000 | OrV |
| *pals-36* | 1.9165 | 0.0007 | OrV |
| *pals-37* | 3.1870 | 0.0000 | OrV |
| *pals-39* | 4.9655 | 0.0000 | OrV |
| *pals-38* | 3.5631 | 0.0000 | OrV |
| *clec-45* | 2.0917 | 0.0017 | OrV |
| *F07E5.9* | 3.8704 | 0.0003 | OrV |
| *F15H10.10* | 8.5427 | 0.0000 | OrV |
| *F15H10.5* | 2.1493 | 0.0096 | OrV |
| *pals-14* | 6.9419 | 0.0000 | OrV |
| *F26D11.13* | 2.8917 | 0.0011 | OrV |
| *F26F2.1* | 4.3526 | 0.0000 | OrV |
| *F26F2.3* | 4.0682 | 0.0000 | OrV |
| *F35E12.10* | 0.9839 | 0.0000 | OrV |
| *F42C5.3* | 6.7559 | 0.0000 | OrV |
| *fbxa-182* | 5.1348 | 0.0000 | OrV |
| *skr-5* | 2.3244 | 0.0004 | OrV |
| *fbxa-188* | 2.0877 | 0.0002 | OrV |
| *sago-2* | 2.3993 | 0.0000 | OrV |
| *F57G4.11* | 6.8899 | 0.0000 | OrV |
| *K10G4.13* | 3.2746 | 0.0000 | OrV |
| *M01G12.7* | 3.6147 | 0.0004 | OrV |
| *lec-8* | 0.5347 | 0.0046 | OrV |
| *T07H8.11* | 4.0827 | 0.0000 | OrV |
| *T08E11.1* | 4.8942 | 0.0000 | OrV |
| *pho-14* | 0.3864 | 0.0195 | OrV |
| *trpl-5* | 6.9092 | 0.0000 | OrV |
| *T19D12.4* | 0.7244 | 0.0066 | OrV |
| *eol-1* | 7.6687 | 0.0000 | OrV |
| *pals-29* | 6.1421 | 0.0001 | OrV |
| *pals-33* | 4.6786 | 0.0000 | OrV |
| *Y105C5A.1269* | 2.2448 | 0.0071 | OrV |
| *lys-1* | 0.2962 | 0.0080 | OrV |
| *lys-3* | 2.7252 | 0.0000 | OrV |
| *Y46G5A.20* | 2.5905 | 0.0000 | OrV |
| *Y47H10A.5* | 1.2888 | 0.0001 | OrV |
| *pals-30* | 4.0054 | 0.0003 | OrV |
| *skr-4* | 1.6118 | 0.0000 | OrV |
| *Y6E2A.5* | 1.1107 | 0.0214 | OrV |
| *Y75B8A.39* | 6.1478 | 0.0000 | OrV |
| *ZC196.3* | 4.7605 | 0.0001 | OrV |
| *droe-8* | 4.9039 | 0.0001 | OrV |
| *ZK896.4* | 1.3209 | 0.0007 | OrV |
| *B0024.4* | 3.4485 | 0.0000 | RSE+OrV |
| *pals-27* | 2.8931 | 0.0000 | RSE+OrV |
| *pals-28* | 3.5487 | 0.0009 | RSE+OrV |
| *valv-1* | 1.5425 | 0.0051 | RSE+OrV |
| *ddn-1* | 4.0133 | 0.0002 | RSE+OrV |
| *B0507.6* | 1.6270 | 0.0041 | RSE+OrV |
| *B0507.7* | 5.3539 | 0.0000 | RSE+OrV |
| *B0507.8* | 7.2159 | 0.0000 | RSE+OrV |
| *C06E4.8* | 1.0714 | 0.0664 | RSE+OrV |
| *fbxa-158* | 2.8131 | 0.0000 | RSE+OrV |
| *math-14* | 0.4198 | 0.3718 | RSE+OrV |
| *C17H1.2* | 2.4910 | 0.0091 | RSE+OrV |
| *pals-2* | 2.7234 | 0.0024 | RSE+OrV |
| *pals-3* | 2.2100 | 0.0071 | RSE+OrV |
| *pals-4* | 5.5307 | 0.0000 | RSE+OrV |
| *pals-5* | 1.1401 | 0.0281 | RSE+OrV |
| *pals-6* | 2.1580 | 0.0000 | RSE+OrV |
| *ppw-1* | 1.4990 | 0.0000 | RSE+OrV |
| *C18H7.11* | 1.3473 | 0.0015 | RSE+OrV |
| *C27D9.2* | 2.1916 | 0.0000 | RSE+OrV |
| *rcs-1* | 0.7868 | 0.0000 | RSE+OrV |
| *pals-32* | 2.3232 | 0.0000 | RSE+OrV |
| *C49C3.9* | 1.4074 | 0.0000 | RSE+OrV |
| *C49C8.8* | 2.0703 | 0.0006 | RSE+OrV |
| *C53A5.9* | 2.9333 | 0.0011 | RSE+OrV |
| *pals-36* | 0.6506 | 0.1406 | RSE+OrV |
| *pals-37* | 1.9554 | 0.0000 | RSE+OrV |
| *pals-39* | 3.3839 | 0.0000 | RSE+OrV |
| *pals-38* | 2.0753 | 0.0008 | RSE+OrV |
| *clec-45* | 2.8625 | 0.0001 | RSE+OrV |
| *F07E5.9* | 1.7542 | 0.0183 | RSE+OrV |
| *F15H10.10* | 5.0567 | 0.0000 | RSE+OrV |
| *F15H10.5* | 0.3314 | 0.4720 | RSE+OrV |
| *pals-14* | 4.8165 | 0.0000 | RSE+OrV |
| *F26D11.13* | 1.3222 | 0.0481 | RSE+OrV |
| *F26F2.1* | 0.7619 | 0.0907 | RSE+OrV |
| *F26F2.3* | 2.0547 | 0.0067 | RSE+OrV |
| *F35E12.10* | 0.8336 | 0.0001 | RSE+OrV |
| *F42C5.3* | 4.3434 | 0.0000 | RSE+OrV |
| *fbxa-182* | 3.6197 | 0.0000 | RSE+OrV |
| *skr-5* | 2.1914 | 0.0017 | RSE+OrV |
| *fbxa-188* | 2.2498 | 0.0001 | RSE+OrV |
| *sago-2* | 1.6314 | 0.0000 | RSE+OrV |
| *F57G4.11* | 3.6887 | 0.0005 | RSE+OrV |
| *K10G4.13* | 2.4051 | 0.0004 | RSE+OrV |
| *M01G12.7* | 1.2951 | 0.0426 | RSE+OrV |
| *lec-8* | 0.2818 | 0.1149 | RSE+OrV |
| *T07H8.11* | 2.2908 | 0.0044 | RSE+OrV |
| *T08E11.1* | 2.9054 | 0.0000 | RSE+OrV |
| *pho-14* | 0.1920 | 0.2264 | RSE+OrV |
| *trpl-5* | 5.4157 | 0.0000 | RSE+OrV |
| *T19D12.4* | 0.6855 | 0.0142 | RSE+OrV |
| *eol-1* | 6.1296 | 0.0000 | RSE+OrV |
| *pals-29* | 2.0683 | 0.0102 | RSE+OrV |
| *pals-33* | 3.2984 | 0.0000 | RSE+OrV |
| *Y105C5A.1269* | 1.1794 | 0.0755 | RSE+OrV |
| *lys-1* | 0.5014 | 0.0000 | RSE+OrV |
| *lys-3* | 2.8374 | 0.0000 | RSE+OrV |
| *Y46G5A.20* | 1.6504 | 0.0000 | RSE+OrV |
| *Y47H10A.5* | 1.6121 | 0.0000 | RSE+OrV |
| *pals-30* | 2.0907 | 0.0116 | RSE+OrV |
| *skr-4* | 0.4027 | 0.1647 | RSE+OrV |
| *Y6E2A.5* | 0.2941 | 0.4030 | RSE+OrV |
| *Y75B8A.39* | 4.6311 | 0.0000 | RSE+OrV |
| *ZC196.3* | 1.7504 | 0.0215 | RSE+OrV |
| *droe-8* | 2.1382 | 0.0112 | RSE+OrV |
| *ZK896.4* | 1.9339 | 0.0000 | RSE+OrV |
| *B0024.4* | 3.2440 | 0.0000 | LSMMG+OrV |
| *pals-27* | 4.5496 | 0.0000 | LSMMG+OrV |
| *pals-28* | 6.6586 | 0.0000 | LSMMG+OrV |
| *valv-1* | 2.2831 | 0.0000 | LSMMG+OrV |
| *ddn-1* | 7.4418 | 0.0000 | LSMMG+OrV |
| *B0507.6* | 3.5367 | 0.0000 | LSMMG+OrV |
| *B0507.7* | 1.6146 | 0.0119 | LSMMG+OrV |
| *B0507.8* | 7.3146 | 0.0000 | LSMMG+OrV |
| *C06E4.8* | 1.3672 | 0.0076 | LSMMG+OrV |
| *fbxa-158* | 2.1654 | 0.0000 | LSMMG+OrV |
| *math-14* | 2.1117 | 0.0092 | LSMMG+OrV |
| *C17H1.2* | 6.0256 | 0.0002 | LSMMG+OrV |
| *pals-2* | 3.4722 | 0.0006 | LSMMG+OrV |
| *pals-3* | 5.2781 | 0.0003 | LSMMG+OrV |
| *pals-4* | 7.0194 | 0.0000 | LSMMG+OrV |
| *pals-5* | 2.6408 | 0.0000 | LSMMG+OrV |
| *pals-6* | 1.0546 | 0.0009 | LSMMG+OrV |
| *ppw-1* | 1.7558 | 0.0000 | LSMMG+OrV |
| *C18H7.11* | 1.4570 | 0.0000 | LSMMG+OrV |
| *C27D9.2* | 2.6878 | 0.0000 | LSMMG+OrV |
| *rcs-1* | 0.4099 | 0.0006 | LSMMG+OrV |
| *pals-32* | 2.6158 | 0.0000 | LSMMG+OrV |
| *C49C3.9* | 1.2955 | 0.0000 | LSMMG+OrV |
| *C49C8.8* | 3.2294 | 0.0000 | LSMMG+OrV |
| *C53A5.9* | 5.4695 | 0.0000 | LSMMG+OrV |
| *pals-36* | 3.9095 | 0.0000 | LSMMG+OrV |
| *pals-37* | 4.3321 | 0.0000 | LSMMG+OrV |
| *pals-39* | 5.0821 | 0.0000 | LSMMG+OrV |
| *pals-38* | 2.2167 | 0.0000 | LSMMG+OrV |
| *clec-45* | 1.6912 | 0.0023 | LSMMG+OrV |
| *F07E5.9* | 1.4293 | 0.0127 | LSMMG+OrV |
| *F15H10.10* | 1.6565 | 0.0115 | LSMMG+OrV |
| *F15H10.5* | 0.4079 | 0.1190 | LSMMG+OrV |
| *pals-14* | 5.6428 | 0.0000 | LSMMG+OrV |
| *F26D11.13* | 2.4971 | 0.0051 | LSMMG+OrV |
| *F26F2.1* | 2.6056 | 0.0000 | LSMMG+OrV |
| *F26F2.3* | 3.8816 | 0.0001 | LSMMG+OrV |
| *F35E12.10* | 1.2357 | 0.0000 | LSMMG+OrV |
| *F42C5.3* | 6.2634 | 0.0000 | LSMMG+OrV |
| *fbxa-182* | 3.1679 | 0.0000 | LSMMG+OrV |
| *skr-5* | 2.2922 | 0.0002 | LSMMG+OrV |
| *fbxa-188* | 0.5575 | 0.0745 | LSMMG+OrV |
| *sago-2* | 2.0621 | 0.0000 | LSMMG+OrV |
| *F57G4.11* | 7.9591 | 0.0000 | LSMMG+OrV |
| *K10G4.13* | 4.5838 | 0.0000 | LSMMG+OrV |
| *M01G12.7* | 1.1789 | 0.0131 | LSMMG+OrV |
| *lec-8* | 0.6713 | 0.0000 | LSMMG+OrV |
| *T07H8.11* | 3.5787 | 0.0000 | LSMMG+OrV |
| *T08E11.1* | 2.5135 | 0.0000 | LSMMG+OrV |
| *pho-14* | 0.9252 | 0.0000 | LSMMG+OrV |
| *trpl-5* | 5.7121 | 0.0000 | LSMMG+OrV |
| *T19D12.4* | 0.3370 | 0.0389 | LSMMG+OrV |
| *eol-1* | 6.0104 | 0.0000 | LSMMG+OrV |
| *pals-29* | 2.4294 | 0.0047 | LSMMG+OrV |
| *pals-33* | 5.8605 | 0.0000 | LSMMG+OrV |
| *Y105C5A.1269* | 5.9255 | 0.0003 | LSMMG+OrV |
| *lys-1* | 0.3746 | 0.0000 | LSMMG+OrV |
| *lys-3* | 3.4107 | 0.0000 | LSMMG+OrV |
| *Y46G5A.20* | 1.4125 | 0.0000 | LSMMG+OrV |
| *Y47H10A.5* | 1.3133 | 0.0000 | LSMMG+OrV |
| *pals-30* | 1.1708 | 0.0220 | LSMMG+OrV |
| *skr-4* | 1.5328 | 0.0000 | LSMMG+OrV |
| *Y6E2A.5* | 2.1747 | 0.0003 | LSMMG+OrV |
| *Y75B8A.39* | 3.9158 | 0.0000 | LSMMG+OrV |
| *ZC196.3* | 0.7977 | 0.0428 | LSMMG+OrV |
| *droe-8* | 2.5894 | 0.0059 | LSMMG+OrV |
| *ZK896.4* | 2.1812 | 0.0000 | LSMMG+OrV |
| *B0024.4* | 0.1218 | 0.6115 | RSE:OrV |
| *pals-27* | -1.9369 | 0.0133 | RSE:OrV |
| *pals-28* | -0.0471 | 0.7302 | RSE:OrV |
| *valv-1* | -0.0807 | 1.0000 | RSE:OrV |
| *ddn-1* | -0.1082 | 0.3672 | RSE:OrV |
| *B0507.6* | 0.0640 | 1.0000 | RSE:OrV |
| *B0507.7* | -0.0006 | 0.2321 | RSE:OrV |
| *B0507.8* | -4.1762 | 0.0000 | RSE:OrV |
| *C06E4.8* | -0.1142 | 1.0000 | RSE:OrV |
| *fbxa-158* | -0.1370 | 0.8036 | RSE:OrV |
| *math-14* | 0.0454 | 1.0000 | RSE:OrV |
| *C17H1.2* | -0.0536 | 1.0000 | RSE:OrV |
| *pals-2* | -0.3050 | 0.0877 | RSE:OrV |
| *pals-3* | -0.0509 | 1.0000 | RSE:OrV |
| *pals-4* | -0.1916 | 0.1556 | RSE:OrV |
| *pals-5* | -1.4313 | 0.0458 | RSE:OrV |
| *pals-6* | -1.1770 | 0.0208 | RSE:OrV |
| *ppw-1* | -1.1011 | 0.0000 | RSE:OrV |
| *C18H7.11* | -0.1283 | 0.8946 | RSE:OrV |
| *C27D9.2* | -0.3563 | 0.4224 | RSE:OrV |
| *rcs-1* | -0.4125 | 0.0387 | RSE:OrV |
| *pals-32* | -0.2582 | 0.4776 | RSE:OrV |
| *C49C3.9* | -0.0241 | 1.0000 | RSE:OrV |
| *C49C8.8* | -0.3144 | 0.3591 | RSE:OrV |
| *C53A5.9* | -0.3491 | 0.0823 | RSE:OrV |
| *pals-36* | 0.0662 | 1.0000 | RSE:OrV |
| *pals-37* | -0.4828 | 0.2261 | RSE:OrV |
| *pals-39* | -0.7543 | 0.0745 | RSE:OrV |
| *pals-38* | -0.2681 | 0.3703 | RSE:OrV |
| *clec-45* | -0.1271 | 0.8070 | RSE:OrV |
| *F07E5.9* | -0.0563 | 1.0000 | RSE:OrV |
| *F15H10.10* | -0.0503 | 0.5945 | RSE:OrV |
| *F15H10.5* | 0.0256 | 1.0000 | RSE:OrV |
| *pals-14* | -2.4091 | 0.0008 | RSE:OrV |
| *F26D11.13* | -0.0329 | 1.0000 | RSE:OrV |
| *F26F2.1* | -1.9878 | 0.0147 | RSE:OrV |
| *F26F2.3* | -0.2238 | 0.2855 | RSE:OrV |
| *F35E12.10* | 0.1436 | 0.5844 | RSE:OrV |
| *F42C5.3* | -3.5234 | 0.0010 | RSE:OrV |
| *fbxa-182* | -1.7942 | 0.0019 | RSE:OrV |
| *skr-5* | -0.1703 | 0.5996 | RSE:OrV |
| *fbxa-188* | -0.2387 | 0.5657 | RSE:OrV |
| *sago-2* | -1.2893 | 0.0000 | RSE:OrV |
| *F57G4.11* | -0.0126 | 1.0000 | RSE:OrV |
| *K10G4.13* | -0.1828 | 0.6253 | RSE:OrV |
| *M01G12.7* | 0.0210 | 1.0000 | RSE:OrV |
| *lec-8* | 0.1055 | 0.8060 | RSE:OrV |
| *T07H8.11* | -0.3659 | 0.0671 | RSE:OrV |
| *T08E11.1* | -1.5548 | 0.0487 | RSE:OrV |
| *pho-14* | 0.0369 | 0.7896 | RSE:OrV |
| *trpl-5* | -2.8657 | 0.0042 | RSE:OrV |
| *T19D12.4* | 0.0553 | 0.7416 | RSE:OrV |
| *eol-1* | -2.9645 | 0.0000 | RSE:OrV |
| *pals-29* | -0.0914 | 0.3618 | RSE:OrV |
| *pals-33* | -0.8323 | 0.1217 | RSE:OrV |
| *Y105C5A.1269* | 0.0028 | 1.0000 | RSE:OrV |
| *lys-1* | 0.0151 | 0.8365 | RSE:OrV |
| *lys-3* | -0.1275 | 0.7350 | RSE:OrV |
| *Y46G5A.20* | -0.2016 | 0.5080 | RSE:OrV |
| *Y47H10A.5* | 0.0733 | 1.0000 | RSE:OrV |
| *pals-30* | -0.2418 | 0.6186 | RSE:OrV |
| *skr-4* | -0.3869 | 0.3054 | RSE:OrV |
| *Y6E2A.5* | -0.0103 | 1.0000 | RSE:OrV |
| *Y75B8A.39* | -0.3453 | 0.1077 | RSE:OrV |
| *ZC196.3* | -0.1124 | 1.0000 | RSE:OrV |
| *droe-8* | -0.1849 | 0.8339 | RSE:OrV |
| *ZK896.4* | 0.0783 | 1.0000 | RSE:OrV |
| *B0024.4* | -0.2132 | 0.3988 | LSMMG:OrV |
| *pals-27* | -0.0857 | 0.7579 | LSMMG:OrV |
| *pals-28* | -0.0168 | 0.7564 | LSMMG:OrV |
| *valv-1* | -0.0253 | 1.0000 | LSMMG:OrV |
| *ddn-1* | -0.1000 | 0.4795 | LSMMG:OrV |
| *B0507.6* | 0.1407 | 0.6719 | LSMMG:OrV |
| *B0507.7* | -9.9149 | 0.0004 | LSMMG:OrV |
| *B0507.8* | -3.6002 | 0.0008 | LSMMG:OrV |
| *C06E4.8* | -0.0621 | 1.0000 | LSMMG:OrV |
| *fbxa-158* | -0.1815 | 0.4711 | LSMMG:OrV |
| *math-14* | -0.0123 | 1.0000 | LSMMG:OrV |
| *C17H1.2* | -0.0056 | 1.0000 | LSMMG:OrV |
| *pals-2* | -0.1636 | 0.2030 | LSMMG:OrV |
| *pals-3* | 0.0367 | 1.0000 | LSMMG:OrV |
| *pals-4* | -0.0983 | 0.3391 | LSMMG:OrV |
| *pals-5* | -0.0489 | 1.0000 | LSMMG:OrV |
| *pals-6* | -0.2170 | 0.4464 | LSMMG:OrV |
| *ppw-1* | -0.5707 | 0.0088 | LSMMG:OrV |
| *C18H7.11* | 0.0206 | 1.0000 | LSMMG:OrV |
| *C27D9.2* | 0.3709 | 0.2681 | LSMMG:OrV |
| *rcs-1* | -0.1324 | 0.6808 | LSMMG:OrV |
| *pals-32* | -0.1725 | 0.4839 | LSMMG:OrV |
| *C49C3.9* | -0.1766 | 0.6408 | LSMMG:OrV |
| *C49C8.8* | -0.3920 | 0.2680 | LSMMG:OrV |
| *C53A5.9* | -0.2804 | 0.1303 | LSMMG:OrV |
| *pals-36* | 0.3057 | 0.2410 | LSMMG:OrV |
| *pals-37* | 1.5857 | 0.0452 | LSMMG:OrV |
| *pals-39* | 0.0199 | 1.0000 | LSMMG:OrV |
| *pals-38* | -0.1114 | 1.0000 | LSMMG:OrV |
| *clec-45* | 0.0019 | 1.0000 | LSMMG:OrV |
| *F07E5.9* | 0.0106 | 1.0000 | LSMMG:OrV |
| *F15H10.10* | -0.0884 | 0.2340 | LSMMG:OrV |
| *F15H10.5* | 0.0230 | 1.0000 | LSMMG:OrV |
| *pals-14* | -0.2321 | 0.2561 | LSMMG:OrV |
| *F26D11.13* | -0.0413 | 1.0000 | LSMMG:OrV |
| *F26F2.1* | -0.0685 | 0.8367 | LSMMG:OrV |
| *F26F2.3* | 0.0373 | 1.0000 | LSMMG:OrV |
| *F35E12.10* | -0.2933 | 0.3311 | LSMMG:OrV |
| *F42C5.3* | -0.0648 | 0.7048 | LSMMG:OrV |
| *fbxa-182* | -0.3984 | 0.1855 | LSMMG:OrV |
| *skr-5* | -0.0488 | 1.0000 | LSMMG:OrV |
| *fbxa-188* | -0.1312 | 0.8921 | LSMMG:OrV |
| *sago-2* | -0.4641 | 0.0667 | LSMMG:OrV |
| *F57G4.11* | 0.0393 | 1.0000 | LSMMG:OrV |
| *K10G4.13* | 0.0751 | 0.8241 | LSMMG:OrV |
| *M01G12.7* | -0.0272 | 1.0000 | LSMMG:OrV |
| *lec-8* | 0.1252 | 0.7503 | LSMMG:OrV |
| *T07H8.11* | -0.1037 | 0.5745 | LSMMG:OrV |
| *T08E11.1* | -0.0888 | 0.7920 | LSMMG:OrV |
| *pho-14* | 0.3837 | 0.1945 | LSMMG:OrV |
| *trpl-5* | -0.0754 | 0.7389 | LSMMG:OrV |
| *T19D12.4* | -0.0148 | 1.0000 | LSMMG:OrV |
| *eol-1* | -2.2948 | 0.0040 | LSMMG:OrV |
| *pals-29* | 0.0051 | 1.0000 | LSMMG:OrV |
| *pals-33* | 0.0882 | 0.6783 | LSMMG:OrV |
| *Y105C5A.1269* | 0.0068 | 1.0000 | LSMMG:OrV |
| *lys-1* | 0.1323 | 0.6012 | LSMMG:OrV |
| *lys-3* | 0.0613 | 0.8797 | LSMMG:OrV |
| *Y46G5A.20* | -0.4129 | 0.1953 | LSMMG:OrV |
| *Y47H10A.5* | -0.0981 | 0.7686 | LSMMG:OrV |
| *pals-30* | -0.0274 | 1.0000 | LSMMG:OrV |
| *skr-4* | -0.1452 | 0.7226 | LSMMG:OrV |
| *Y6E2A.5* | 0.0763 | 0.8807 | LSMMG:OrV |
| *Y75B8A.39* | -0.3944 | 0.1001 | LSMMG:OrV |
| *ZC196.3* | -0.1728 | 0.6461 | LSMMG:OrV |
| *droe-8* | -0.0619 | 1.0000 | LSMMG:OrV |
| *ZK896.4* | 0.5057 | 0.3555 | LSMMG:OrV |
| *B0024.4* | 4.3684 | 0.0000 | RSE+OrV vs RSE |
| *pals-27* | 2.1498 | 0.0000 | RSE+OrV vs RSE |
| *pals-28* | 4.8856 | 0.0006 | RSE+OrV vs RSE |
| *valv-1* | 2.2222 | 0.0000 | RSE+OrV vs RSE |
| *ddn-1* | 5.9562 | 0.0000 | RSE+OrV vs RSE |
| *B0507.6* | 3.7550 | 0.0000 | RSE+OrV vs RSE |
| *B0507.7* | 5.7774 | 0.0000 | RSE+OrV vs RSE |
| *B0507.8* | 5.4544 | 0.0000 | RSE+OrV vs RSE |
| *C06E4.8* | 2.1345 | 0.0063 | RSE+OrV vs RSE |
| *fbxa-158* | 3.1780 | 0.0000 | RSE+OrV vs RSE |
| *math-14* | 4.6775 | 0.0007 | RSE+OrV vs RSE |
| *C17H1.2* | 2.8990 | 0.0419 | RSE+OrV vs RSE |
| *pals-2* | 2.8003 | 0.0002 | RSE+OrV vs RSE |
| *pals-3* | 3.9284 | 0.0023 | RSE+OrV vs RSE |
| *pals-4* | 5.3800 | 0.0000 | RSE+OrV vs RSE |
| *pals-5* | 0.6450 | 0.0489 | RSE+OrV vs RSE |
| *pals-6* | 2.4131 | 0.0000 | RSE+OrV vs RSE |
| *ppw-1* | 1.0572 | 0.0000 | RSE+OrV vs RSE |
| *C18H7.11* | 0.7739 | 0.0062 | RSE+OrV vs RSE |
| *C27D9.2* | 0.8890 | 0.0012 | RSE+OrV vs RSE |
| *rcs-1* | 0.1810 | 0.0389 | RSE+OrV vs RSE |
| *pals-32* | 3.3363 | 0.0000 | RSE+OrV vs RSE |
| *C49C3.9* | 1.0048 | 0.0000 | RSE+OrV vs RSE |
| *C49C8.8* | 2.3932 | 0.0000 | RSE+OrV vs RSE |
| *C53A5.9* | 3.2120 | 0.0001 | RSE+OrV vs RSE |
| *pals-36* | 2.8803 | 0.0000 | RSE+OrV vs RSE |
| *pals-37* | 2.3577 | 0.0000 | RSE+OrV vs RSE |
| *pals-39* | 3.7643 | 0.0000 | RSE+OrV vs RSE |
| *pals-38* | 2.5287 | 0.0000 | RSE+OrV vs RSE |
| *clec-45* | 1.9617 | 0.0000 | RSE+OrV vs RSE |
| *F07E5.9* | 4.0026 | 0.0000 | RSE+OrV vs RSE |
| *F15H10.10* | 5.9092 | 0.0000 | RSE+OrV vs RSE |
| *F15H10.5* | 4.6835 | 0.0035 | RSE+OrV vs RSE |
| *pals-14* | 4.2563 | 0.0000 | RSE+OrV vs RSE |
| *F26D11.13* | 2.6662 | 0.0353 | RSE+OrV vs RSE |
| *F26F2.1* | 1.9240 | 0.0002 | RSE+OrV vs RSE |
| *F26F2.3* | 1.7082 | 0.0176 | RSE+OrV vs RSE |
| *F35E12.10* | 1.2218 | 0.0000 | RSE+OrV vs RSE |
| *F42C5.3* | 3.0026 | 0.0000 | RSE+OrV vs RSE |
| *fbxa-182* | 3.0824 | 0.0000 | RSE+OrV vs RSE |
| *skr-5* | 1.2654 | 0.0148 | RSE+OrV vs RSE |
| *fbxa-188* | 1.3993 | 0.0006 | RSE+OrV vs RSE |
| *sago-2* | 1.0557 | 0.0000 | RSE+OrV vs RSE |
| *F57G4.11* | 6.0117 | 0.0000 | RSE+OrV vs RSE |
| *K10G4.13* | 2.6110 | 0.0000 | RSE+OrV vs RSE |
| *M01G12.7* | 4.9309 | 0.0001 | RSE+OrV vs RSE |
| *lec-8* | 0.8290 | 0.0000 | RSE+OrV vs RSE |
| *T07H8.11* | 0.8028 | 0.0458 | RSE+OrV vs RSE |
| *T08E11.1* | 2.8520 | 0.0000 | RSE+OrV vs RSE |
| *pho-14* | 0.4927 | 0.0001 | RSE+OrV vs RSE |
| *trpl-5* | 3.7306 | 0.0000 | RSE+OrV vs RSE |
| *T19D12.4* | 1.0325 | 0.0000 | RSE+OrV vs RSE |
| *eol-1* | 4.5525 | 0.0000 | RSE+OrV vs RSE |
| *pals-29* | 3.2428 | 0.0048 | RSE+OrV vs RSE |
| *pals-33* | 3.1575 | 0.0000 | RSE+OrV vs RSE |
| *Y105C5A.1269* | 3.9876 | 0.0104 | RSE+OrV vs RSE |
| *lys-1* | 0.3116 | 0.0000 | RSE+OrV vs RSE |
| *lys-3* | 2.5524 | 0.0000 | RSE+OrV vs RSE |
| *Y46G5A.20* | 2.2113 | 0.0000 | RSE+OrV vs RSE |
| *Y47H10A.5* | 1.6220 | 0.0000 | RSE+OrV vs RSE |
| *pals-30* | 2.0681 | 0.0021 | RSE+OrV vs RSE |
| *skr-4* | 0.7665 | 0.0071 | RSE+OrV vs RSE |
| *Y6E2A.5* | 1.5829 | 0.0031 | RSE+OrV vs RSE |
| *Y75B8A.39* | 3.5841 | 0.0000 | RSE+OrV vs RSE |
| *ZC196.3* | 3.1052 | 0.0088 | RSE+OrV vs RSE |
| *droe-8* | 2.8567 | 0.0006 | RSE+OrV vs RSE |
| *ZK896.4* | 1.8307 | 0.0000 | RSE+OrV vs RSE |
| *B0024.4* | 3.0222 | 0.0000 | LSMMG+OrV vs LSMMG |
| *pals-27* | 3.7949 | 0.0000 | LSMMG+OrV vs LSMMG |
| *pals-28* | 4.6122 | 0.0052 | LSMMG+OrV vs LSMMG |
| *valv-1* | 2.5123 | 0.0000 | LSMMG+OrV vs LSMMG |
| *ddn-1* | 6.0627 | 0.0000 | LSMMG+OrV vs LSMMG |
| *B0507.6* | 4.3807 | 0.0000 | LSMMG+OrV vs LSMMG |
| *B0507.7* | 2.6559 | 0.0010 | LSMMG+OrV vs LSMMG |
| *B0507.8* | 5.8327 | 0.0000 | LSMMG+OrV vs LSMMG |
| *C06E4.8* | 2.4173 | 0.0017 | LSMMG+OrV vs LSMMG |
| *fbxa-158* | 2.8374 | 0.0000 | LSMMG+OrV vs LSMMG |
| *math-14* | 4.1730 | 0.0003 | LSMMG+OrV vs LSMMG |
| *C17H1.2* | 5.0442 | 0.0030 | LSMMG+OrV vs LSMMG |
| *pals-2* | 2.9526 | 0.0010 | LSMMG+OrV vs LSMMG |
| *pals-3* | 6.8984 | 0.0000 | LSMMG+OrV vs LSMMG |
| *pals-4* | 6.0184 | 0.0000 | LSMMG+OrV vs LSMMG |
| *pals-5* | 2.9605 | 0.0000 | LSMMG+OrV vs LSMMG |
| *pals-6* | 3.1121 | 0.0000 | LSMMG+OrV vs LSMMG |
| *ppw-1* | 1.5581 | 0.0000 | LSMMG+OrV vs LSMMG |
| *C18H7.11* | 1.2913 | 0.0000 | LSMMG+OrV vs LSMMG |
| *C27D9.2* | 3.0509 | 0.0000 | LSMMG+OrV vs LSMMG |
| *rcs-1* | 0.5546 | 0.0000 | LSMMG+OrV vs LSMMG |
| *pals-32* | 3.3679 | 0.0000 | LSMMG+OrV vs LSMMG |
| *C49C3.9* | 0.5516 | 0.0099 | LSMMG+OrV vs LSMMG |
| *C49C8.8* | 1.8491 | 0.0002 | LSMMG+OrV vs LSMMG |
| *C53A5.9* | 3.5349 | 0.0000 | LSMMG+OrV vs LSMMG |
| *pals-36* | 4.9290 | 0.0000 | LSMMG+OrV vs LSMMG |
| *pals-37* | 5.5411 | 0.0000 | LSMMG+OrV vs LSMMG |
| *pals-39* | 5.0995 | 0.0000 | LSMMG+OrV vs LSMMG |
| *pals-38* | 2.8881 | 0.0000 | LSMMG+OrV vs LSMMG |
| *clec-45* | 3.0366 | 0.0000 | LSMMG+OrV vs LSMMG |
| *F07E5.9* | 5.3089 | 0.0000 | LSMMG+OrV vs LSMMG |
| *F15H10.10* | 4.8217 | 0.0000 | LSMMG+OrV vs LSMMG |
| *F15H10.5* | 4.8671 | 0.0038 | LSMMG+OrV vs LSMMG |
| *pals-14* | 5.4029 | 0.0000 | LSMMG+OrV vs LSMMG |
| *F26D11.13* | 1.1340 | 0.0280 | LSMMG+OrV vs LSMMG |
| *F26F2.1* | 3.9894 | 0.0000 | LSMMG+OrV vs LSMMG |
| *F26F2.3* | 5.0874 | 0.0001 | LSMMG+OrV vs LSMMG |
| *F35E12.10* | 0.5996 | 0.0000 | LSMMG+OrV vs LSMMG |
| *F42C5.3* | 5.5384 | 0.0000 | LSMMG+OrV vs LSMMG |
| *fbxa-182* | 3.8578 | 0.0000 | LSMMG+OrV vs LSMMG |
| *skr-5* | 2.1173 | 0.0036 | LSMMG+OrV vs LSMMG |
| *fbxa-188* | 1.2476 | 0.0052 | LSMMG+OrV vs LSMMG |
| *sago-2* | 1.8268 | 0.0000 | LSMMG+OrV vs LSMMG |
| *F57G4.11* | 9.1343 | 0.0000 | LSMMG+OrV vs LSMMG |
| *K10G4.13* | 4.6981 | 0.0000 | LSMMG+OrV vs LSMMG |
| *M01G12.7* | 3.8651 | 0.0014 | LSMMG+OrV vs LSMMG |
| *lec-8* | 0.8015 | 0.0000 | LSMMG+OrV vs LSMMG |
| *T07H8.11* | 3.0212 | 0.0003 | LSMMG+OrV vs LSMMG |
| *T08E11.1* | 4.1962 | 0.0000 | LSMMG+OrV vs LSMMG |
| *pho-14* | 0.9604 | 0.0000 | LSMMG+OrV vs LSMMG |
| *trpl-5* | 5.6542 | 0.0000 | LSMMG+OrV vs LSMMG |
| *T19D12.4* | 0.6736 | 0.0010 | LSMMG+OrV vs LSMMG |
| *eol-1* | 5.0308 | 0.0000 | LSMMG+OrV vs LSMMG |
| *pals-29* | 5.7518 | 0.0010 | LSMMG+OrV vs LSMMG |
| *pals-33* | 5.7529 | 0.0000 | LSMMG+OrV vs LSMMG |
| *Y105C5A.1269* | 1.9607 | 0.0259 | LSMMG+OrV vs LSMMG |
| *lys-1* | 0.4795 | 0.0000 | LSMMG+OrV vs LSMMG |
| *lys-3* | 3.0320 | 0.0000 | LSMMG+OrV vs LSMMG |
| *Y46G5A.20* | 1.7977 | 0.0000 | LSMMG+OrV vs LSMMG |
| *Y47H10A.5* | 0.8767 | 0.0013 | LSMMG+OrV vs LSMMG |
| *pals-30* | 4.1614 | 0.0005 | LSMMG+OrV vs LSMMG |
| *skr-4* | 1.1627 | 0.0001 | LSMMG+OrV vs LSMMG |
| *Y6E2A.5* | 2.5609 | 0.0001 | LSMMG+OrV vs LSMMG |
| *Y75B8A.39* | 2.8354 | 0.0000 | LSMMG+OrV vs LSMMG |
| *ZC196.3* | 1.2847 | 0.0175 | LSMMG+OrV vs LSMMG |
| *droe-8* | 2.4417 | 0.0165 | LSMMG+OrV vs LSMMG |
| *ZK896.4* | 2.8721 | 0.0000 | LSMMG+OrV vs LSMMG |
| *lips-10* | -3.1631 | 0.0000 | RSE |
| *F47E1.2* | -2.9323 | 0.0000 | RSE |
| *lips-17* | -2.4729 | 0.0000 | RSE |
| *ugt-5* | -0.1902 | 0.2430 | RSE |
| *ZK1193.2* | 0.0888 | 0.4725 | RSE |
| *tps-1* | -1.4760 | 0.0000 | RSE |
| *COX1* | -0.0383 | 0.7667 | RSE |
| *lips-10* | -0.3820 | 0.0003 | LSMMG |
| *F47E1.2* | 0.1329 | 0.2212 | LSMMG |
| *lips-17* | -0.2455 | 0.0299 | LSMMG |
| *ugt-5* | -0.0842 | 0.4273 | LSMMG |
| *ZK1193.2* | -0.0779 | 0.4031 | LSMMG |
| *tps-1* | -0.0782 | 0.3407 | LSMMG |
| *COX1* | 0.0215 | 0.8033 | LSMMG |
| *lips-10* | -1.5215 | 0.0000 | OrV |
| *F47E1.2* | -2.2365 | 0.0000 | OrV |
| *lips-17* | -1.8446 | 0.0000 | OrV |
| *ugt-5* | -0.4982 | 0.0018 | OrV |
| *ZK1193.2* | -0.6473 | 0.0000 | OrV |
| *tps-1* | -1.3661 | 0.0000 | OrV |
| *COX1* | -0.3201 | 0.0289 | OrV |
| *lips-10* | -3.6862 | 0.0000 | RSE+OrV |
| *F47E1.2* | -3.4964 | 0.0000 | RSE+OrV |
| *lips-17* | -3.0653 | 0.0000 | RSE+OrV |
| *ugt-5* | -0.5192 | 0.0022 | RSE+OrV |
| *ZK1193.2* | -0.1076 | 0.4991 | RSE+OrV |
| *tps-1* | -1.8317 | 0.0000 | RSE+OrV |
| *COX1* | -0.2738 | 0.0600 | RSE+OrV |
| *lips-10* | -0.7388 | 0.0000 | LSMMG+OrV |
| *F47E1.2* | -0.2754 | 0.0554 | LSMMG+OrV |
| *lips-17* | -1.0705 | 0.0000 | LSMMG+OrV |
| *ugt-5* | -0.6637 | 0.0000 | LSMMG+OrV |
| *ZK1193.2* | -0.4593 | 0.0001 | LSMMG+OrV |
| *tps-1* | -0.3359 | 0.0002 | LSMMG+OrV |
| *COX1* | -0.2867 | 0.0144 | LSMMG+OrV |
| *lips-10* | 0.9953 | 0.0000 | RSE:OrV |
| *F47E1.2* | 1.6724 | 0.0000 | RSE:OrV |
| *lips-17* | 1.2203 | 0.0000 | RSE:OrV |
| *ugt-5* | 0.1970 | 0.5244 | RSE:OrV |
| *ZK1193.2* | 0.3595 | 0.0708 | RSE:OrV |
| *tps-1* | 1.0002 | 0.0000 | RSE:OrV |
| *COX1* | 0.0722 | 0.6832 | RSE:OrV |
| *lips-10* | 1.1539 | 0.0000 | LSMMG:OrV |
| *F47E1.2* | 1.4643 | 0.0000 | LSMMG:OrV |
| *lips-17* | 1.0084 | 0.0003 | LSMMG:OrV |
| *ugt-5* | 0.0118 | 1.0000 | LSMMG:OrV |
| *ZK1193.2* | 0.2343 | 0.3422 | LSMMG:OrV |
| *tps-1* | 1.0839 | 0.0000 | LSMMG:OrV |
| *COX1* | -0.0033 | 1.0000 | LSMMG:OrV |
| *lips-10* | -0.4380 | 0.0000 | RSE+OrV vs RSE |
| *F47E1.2* | -0.2469 | 0.0311 | RSE+OrV vs RSE |
| *lips-17* | -0.3521 | 0.0156 | RSE+OrV vs RSE |
| *ugt-5* | -0.1881 | 0.0422 | RSE+OrV vs RSE |
| *ZK1193.2* | -0.1740 | 0.0422 | RSE+OrV vs RSE |
| *tps-1* | -0.2894 | 0.0003 | RSE+OrV vs RSE |
| *COX1* | -0.1873 | 0.0250 | RSE+OrV vs RSE |
| *lips-10* | -0.1859 | 0.0229 | LSMMG+OrV vs LSMMG |
| *F47E1.2* | -0.5573 | 0.0017 | LSMMG+OrV vs LSMMG |
| *lips-17* | -0.6651 | 0.0002 | LSMMG+OrV vs LSMMG |
| *ugt-5* | -0.4727 | 0.0056 | LSMMG+OrV vs LSMMG |
| *ZK1193.2* | -0.2813 | 0.0116 | LSMMG+OrV vs LSMMG |
| *tps-1* | -0.1712 | 0.0171 | LSMMG+OrV vs LSMMG |
| *COX1* | -0.2768 | 0.0057 | LSMMG+OrV vs LSMMG |

| **Table S3.** List of tested genes across conditions that were previously described to play a proviral role during OrV infection. | | | |  |
| --- | --- | --- | --- | --- |
| **Gene** | **Role** | **Evidence summary** | **Reference** | |
| *sid-3* | proviral (required for entry/early step) | Mutants strongly defective for OrV replication. Part of conserved TNK2-WASP-NCK1 pathway. | (1) | |
| *viro-2* | proviral (required for entry/early step) | Mapped from virus-induced reporter OFF mutants; required for efficient OrV infection. | (1) | |
| *nck-1* | proviral (required for entry/early step) | RNAi knockdown and mutants reduce OrV infection; part of conserved pathway with *sid-3* and *viro-2*. | (1) | |
| *drl-1* | proviral (affects prereplication/entry stage) | Essential for OrV infection; may affect an entry/prereplication step. | (2) | |
| *hipr-1* | proviral (early stage of infection) | *hipr-1* mutants show defect in OrV infection; human ANTH domain rescues phenotype indicating conserved function. | (3) | |
| *alg-1* | proviral (required for RNA replication) | Mutation of argonaute-like gene *alg-1* causes >10,000-fold reduction in OrV RNA; slicer activity not required. | (4) | |
| *sbp-1* | Master regulator of lipid biosynthesis (SREBP ortholog) | loss-of-function *sbp-1(ep79)* causes major lipid depletion and strongly reduces OrV RNA; replication is rescued by restoring downstream lipids or by lowering Zn. | (5) | |
| *fat-6;fat-7* | Δ9 fatty-acid desaturases (enzymes producing unsaturated fatty acids downstream of SBP-1) | RNAi or mutants of *fat-6;fat-7* reduce OrV RNA; dietary supplementation with lipids downstream of *fat-6;fat-7* rescues viral replication in *sbp-1* mutants. | (5) | |
| *elo-5;elo-6* | Fatty-acid elongases (elongate FAs in *sbp-1* regulated branches) | *elo-5* and *elo-6* knockdown/mutation decrease OrV RNA, implicating these elongases in providing lipids required for viral replication; effects shown by RNAi and mutant challenge experiments. | (5) | |
| 1. Jiang H, Chen K, Sandoval LE, Leung C, Wang D. 2017. An evolutionarily conserved pathway essential for Orsay virus infection of *Caenorhabditis elegans*. mBio 8:e00940-17.  2. Sandoval LE, Jiang H, Wang D. 2019. The dietary restriction-like gene *drl-1*, which encodes a putative serine/threonine kinase, is essential for Orsay virus infection in *Caenorhabditis elegans*. J Virol 93:e01400-18.  3. Jiang H, Sandoval del Prado LE. Leung C, Wang D. 2020. Huntingtin-interacting protein family members have a conserved pro-viral function from *Caenorhabditis elegans* to humans. Proc Natl Acad Sci USA 117:22462-22472.  4. Cubillas C *et al*. 2023. The *alg-1* gene is necessary for Orsay virus replication in *Caenorhabditis elegans*. J Virol 97:e00065-23.  5. Casorla-Perez LA *et al*. 2022. Orsay virus infection of *Caenorhabditis elegans* is modulated by zinc and dependent on lipids. J Virol 96:e01211-22. | | | | |
